# A Tutorial for Information Theory in Neuroscience

**DOI:** 10.1523/ENEURO.0052-18.2018

**Published:** 2018-09-11

**Authors:** Nicholas M. Timme, Christopher Lapish

**Affiliations:** Department of Psychology, Indiana University – Purdue University Indianapolis, 402 N. Blackford St, Indianapolis, IN 46202

**Keywords:** Information flow, information theory, mutual information, neural computation, neural encoding, transfer entropy

## Abstract

Understanding how neural systems integrate, encode, and compute information is central to understanding brain function. Frequently, data from neuroscience experiments are multivariate, the interactions between the variables are nonlinear, and the landscape of hypothesized or possible interactions between variables is extremely broad. Information theory is well suited to address these types of data, as it possesses multivariate analysis tools, it can be applied to many different types of data, it can capture nonlinear interactions, and it does not require assumptions about the structure of the underlying data (i.e., it is model independent). In this article, we walk through the mathematics of information theory along with common logistical problems associated with data type, data binning, data quantity requirements, bias, and significance testing. Next, we analyze models inspired by canonical neuroscience experiments to improve understanding and demonstrate the strengths of information theory analyses. To facilitate the use of information theory analyses, and an understanding of how these analyses are implemented, we also provide a free MATLAB software package that can be applied to a wide range of data from neuroscience experiments, as well as from other fields of study.

## Significance Statement

A primary function of the brain is to process and store information. Therefore, it makes sense to analyze the behavior of the brain using information theory, a statistical tool especially designed to quantify information. Furthermore, given improvements in data-gathering techniques, the power of information theory to analyze large, complex data sets is particularly relevant. In this tutorial, we provide a thorough introduction to information theory and how it can be applied to data gathered from the brain. Our primary audience for this tutorial is researchers new to information theory. We provide numerous intuitive examples including small abstract systems, small and large brain circuits, systems from famous neuroscience experiments, and free software to implement all calculations and models presented herein.

## Introduction

The brain has numerous levels of interaction ranging from gene networks that control cell function to neural circuits that control behavior. While the study of each of these levels requires highly specialized data acquisition approaches, they are similar in that they all require the assessment of interactions among numerous variables that fluctuate over time. Improved data acquisition and computing technologies have produced more complex and exhaustive insights into neural processing. Data from neuroscience experiments are increasingly multivariate, such as simultaneous recordings of many neurons or voxels. Moreover, experiments that simultaneously acquire data of different types are common. For instance, an awake behaving *in vivo* calcium imaging experiment with a stimulus and a behavior possesses at least three distinct types of data (physiologic, behavioral, and stimulation data). This improvement in data quality and scale presents a challenge: how can sometimes subtle, yet important, interactions among variables and the computations they perform be optimally captured? Answering this question is further complicated by the fact that data from neuroscience experiments are frequently noisy and represent systems with nonlinear relationships. Finally, it is often very difficult to develop hypotheses for rules or models that govern the interactions between the numerous variables in the data that can be tested in a clear and straightforward fashion. Information theory (Cover and Thomas, 2006; Stone, 2018) represents a valuable tool to address these increasingly common data analysis concerns.

Because of its general applicability, information theory has been widely used in neuroscience (Rieke et al., 1997; Borst and Theunissen, 1999; Victor, 2006; Quiroga and Panzeri, 2009; Dimitrov et al., 2011; Timme et al., 2014a; Wibral et al., 2014a,b). For instance, research has focused on analyses of electroencephalography (EEG), magnetoencephalography (MEG), and functional MRI (fMRI) data (Jeong et al., 2001; Lizier et al., 2011; Vicente et al., 2011). Research has also focused on trial-based data (Wollstadt et al., 2014; Gomez-Herraro et al., 2015; Asaad et al., 2017) and single-trial time-averaged analyses (Wibral et al., 2013; Timme et al., 2014b, 2016; Nigam et al., 2016). Two particular areas of interest include studies of connectivity (Honey et al., 2007; Ito et al., 2011; Timme et al., 2014b, 2016; Nigam et al., 2016; Wollstadt et al., 2017) and sensory encoding (Bialek et al., 1991; DeWeese and Meister, 1999; Brenner et al., 2000; Panzeri et al., 2001; Butts, 2003; Butts et al., 2007). Throughout these analyses, researchers have used continuous data (e.g., BOLD signal and voltage) as well as discrete data (e.g., action potentials). These studies have produced a wide range of important and interesting results that have contributed to the advancement of neuroscience.

In this article, we present a general overview of commonly used information theory metrics along with applications to several neuroscience systems. We sought to provide an easily accessible discussion of information theory for researchers new to the field. We provide numerous citations to more advanced reviews and especially important texts to address topics not covered herein, though we do not present a wide review of all the neuroscience experiments that have used information theory.

The discussion of the mathematics surrounding information theory includes sections on probability distributions (including issues surrounding binning, continuous versus discrete data, and single trials versus trial-based data), numerous information theory measures (along with simple general examples to gain intuition), significance testing, the methods to provide models that describe the interactions of the underlying variables, and the simulations used in the creation of the neuroscience examples. To foster continued use of information theory in neuroscience, we also include a MATLAB software package (the Neuroscience Information Theory Toolbox; https://github.com/nmtimme/Neuroscience-Information-Theory-Toolbox).

Finally, we apply information theory analyses to simulated data generated from simple neuron models in a wide variety of circumstances, including numerous simple circuit models, a model of sensory habituation, a model of movement direction encoding by primary motor cortex neurons, a model of location encoding by place cells, and a model of light stimulus response by center-surround retinal ganglion cells. These simulations focused on neural spiking data. However, the information theory analyses discussed herein and the accompanying software can be applied to a wide variety of neuroscience data including blood oxygen level–dependent (BOLD) signal data from fMRI studies, fluorescence data from calcium imaging studies, voltage signals from extracellular, EEG, or MEG studies, animal behavior data, genetic data, or molecular concentrations.

## Materials and Methods

### The big-picture view of information theory

#### What is information?

What, specifically, do we mean when we talk about “information”? Clearly, information can mean many different things in different contexts (Adriaans, 2012), but in neuroscience, information is frequently invoked when discussing information encoding (i.e., stimulus encoding), information processing (i.e., decision-making), and information storage (i.e., memory). In our interactions with other scientists, we have found that a lack of understanding about the meaning of information is often the most significant impediment to using information theory analyses. In large part, this article was written with clarifying this issue in mind. If you have ever found yourself saying things like, “They measured an information of 0.05 bits. What does that mean?”, we hope you find some satisfying resolution in this article.

In information theory, one variable provides information about another variable when knowledge of the first, on average, reduces uncertainty in the second (Cover and Thomas, 2006). (To be more precise, this is called “mutual information,” but we’ll stick with “information” for now.) For instance, suppose you and I meet at a conference. I turn my back to you and flip a fair coin. Then, you ask me, “Did the coin come up heads?” I truthfully tell you, “Yes.” The word I said reduced your uncertainty in the state of the coin (you didn’t know the result of the coin flip, but now you do), so my message contained information about (encoded) the coin’s state. It turns out that because the state of the coin has two equally likely values (50% chance heads and 50% chance tails), my message contained 1 bit of information. As we’ll see below, bits can be thought of as the average number of yes/no questions required to ascertain the value of a variable. In the case of a coin, a yes/no answer to one question from you (e.g., “Did the coin come up heads?”) will allow you to determine the state of the coin.

Similar to a coin flip, in a neuroscience context, we can measure how much information a neural variable (analogous to my message) contains about a stimulus (analogous to the coin flip result), for instance. Unsurprisingly, information theory analyses can become far more complex (e.g., what if I lie to you sometimes, what if there are two coins, or what if we do not have whole bits?), but the crucial point is that information is the reduction in uncertainty. If one variable provides information about another, knowing the state of one variable on average allows you to better predict the state of the other than you would have if you did not know the first variable.

#### Why use information theory?

What makes information theory a useful analysis tool in neuroscience? First, information theory is model independent. In other words, it is not necessary to hypothesize a specific structure to the interactions between variables in a data set to use information theory. When applying an information theoretic measure to data, the result is not a parameter in a model (e.g., synaptic strength), but rather a number that quantifies some relationship within the data. The model-free character of information theory allows a much wider range of interactions and phenomena to be quantified than could be achieved with a model-dependent approach that is limited by the assumed model. To be clear, information theoretic analyses typically require some assumptions about the data. Frequently, it is assumed that the system is not changing throughout observations (e.g., a neuron that encodes a stimulus at the beginning of the experiment will do the same at the end), though allowances can be made to accommodate such changes. Furthermore, parameters such as bin sizes involved in discretization are chosen in the analysis, and the choice of these parameter can affect final results. However, the underlying relationships between the variables under study need not follow a predefined model.

Second, information theory can be applied to any mixture of data types. Information theory is capable of producing meaningful measurements when the data are originally any combination of, for instance, action potentials, BOLD signals, voltage values, animal positions, stimulus light position, drug dosages, or lever presses. The original data can be continuous or discrete, and the data can be gathered over a single trial or via repeated trials. Furthermore, the original data can be first processed via a wavelet transform (Hramov et al., 2015) or a dimensionality reduction technique (Cunningham and Yu, 2014), for instance, and then fed into an information theory analysis. This is especially important for recent studies of interactions across different hierarchical levels within the brain (e.g., interneuron to inter–brain region communication).

Third, information theory is capable of detecting linear and nonlinear interactions. Given the prevalence of nonlinear phenomena in neuroscience (the action potential being a central example), this ability is especially important.

Fourth, information theory is naturally multivariate. It possesses several metrics designed to quantify the behavior of systems with any number of variables, from single-variable systems to systems with very large numbers of variables (at least in principle). The information theory measures that are easiest to understand and apply to experimental data tend to involve one to three variables, so we will primarily discuss multivariate measures up to three variables in this tutorial. Importantly, given recent advances in recording large numbers of neural variables (e.g., large multielectrode arrays, calcium imaging, fMRI, etc.), the multivariate analysis capabilities of information theory are especially relevant.

Fifth, information theory produces results in general units of bits. This facilitates straightforward comparisons between cells, brain regions, animal strains, tasks, models, or subjects, though possible biases must be considered in such comparisons. This ability to measure effects in bits allows for direct evaluations of effect sizes.

As with any analysis, information theory also possesses some disadvantages in comparison to other methods (see *What can information theory tell you?*). However, the ability of information theory to detect a wide range of interactions and structure in large complicated systems is especially valuable at this time in neuroscience. Therefore, we feel that additional resources, such as this tutorial and the accompanying software, are critical to communicate the strengths and weaknesses of information theory to the neuroscience community.

#### What can information theory tell you?

While information theory is a powerful tool for highlighting interesting interactions in a wide variety of systems, it is important to distinguish the types of questions information theory can and cannot answer. The result of an information theory analysis is a number or set of numbers that can describe many aspects of the system under study. For instance, it is possible to quantify the uncertainty of one or more variables and dependencies between variables, as well as the influence of one or more variables on one or more other variables (Cover and Thomas, 2006). Often, these sorts of analyses are valuable because they can quantify phenomena like encoding (e.g., how much information a neuron provides; Bialek et al., 1991; Brenner et al., 2000) and complex encoding relationships (e.g., how much information neurons A and B provide together; Bettencourt et al., 2008; Timme et al., 2016).

Though the results of information theory analyses are valuable for certain types of questions, information theory analyses are not capable of producing models that describe how the system works. For instance, if an information theory analysis yields a result that neuron A and neuron B share 0.05 bits of information, little is learned about this system of neurons beyond the fact that their activities are related to a certain extent. For instance, the information theory analysis does not tell us if the neurons are related via an excitatory or inhibitory interaction. Crucially, information theory can be used to restrict the space of possible models (e.g., various information theory quantities can be used to answer questions related to the direction of interactions between variables: does neuron A drive neuron B or vice versa?), but information theory does not produce a model in terms of the original variables that were fed into the information theory analysis. Information theory does not produce a model with spike times, voltage values, spike rates, or any other physical quantity.

We feel this point is of great practical importance when designing an information theory analysis. If you want to build a model, information theory will be a helpful tool to organize your model by, for instance, limiting which variables are in your model and giving you an idea of what sorts of interactions will be necessary between the variables. However, if you desire a model, additional model-building and -fitting techniques will be necessary, because an information theory analysis will not eliminate all possible models (James and Crutchfield, 2017). In this article, though we will briefly discuss some of these models, we will not focus on model building because it is highly system specific. If your ultimate goal is model building, our goal in this article is to provide you with information theory tools to help you guide your model building.

### Probability distributions and initial analysis steps

#### What is a probability distribution?

Fundamentally, information theory makes statements about the shapes of probability distributions. Thus, before discussing information theory measures, it is first necessary to discuss probability distributions. To establish intuition, we will primarily focus on examples involving coins and abstract variables because these are more straightforward and the math often works out to be more aesthetically pleasing. As we will see, neurons rarely encode 1 bit of information precisely, but systems of coins can easily be made to produce whole bits of information. However, the tools we will discuss can just as easily be applied to neuroscience data, as we will see below.

A probability distribution is simply a distribution that describes the likelihood of certain outcomes of a random variable or group of variables. Probability distributions [notated as p(A)] can be discrete (“probability mass function”) or continuous (“probability density function”). They can describe one variable or multiple variables (referred to as a “joint probability distribution”). For instance, if we call p the probability mass function for a fair coin (c is the state of the coin), then $p\left(c=heads\right)=0.5$ and $p\left(c=tails\right)=0.5$ because a fair coin will land heads side up 50% the time when it is flipped and tails the other 50% the time. We use a discrete probability distribution to describe a flip of a coin because its states are discrete. It can only be heads or tails, not some proportion of heads and tails. A biased coin might have a probability distribution of $p\left(c=heads\right)=0.75$ and $p\left(c=tails\right)=0.25$, indicating that 75% of the time the coin lands with the heads side up and only 25% of the time lands with the tails side up. Note that the sum of the possible states in a discrete probability distribution and the integral of the possible values in a continuous probability distribution must be 1.

We can describe systems of more than one variable using a joint probability distribution. If we had two coins ($c_{1}$ and $c_{2}$) that were independent and fair, then the joint probability distribution (notated as p(A,B) would be $p\left(c_{1},c_{2}\right)=0.25$ for all four possible combinations of heads and tails. In this case, because the coins are independent, $p\left(c_{1},c_{2}\right)=p\left(c_{1}\right)p\left(c_{2}\right)$. In cases with dependent variables, this relationship does not hold.

In addition to joint probability distributions, other types of probability distributions are frequently useful in information theory analyses. A marginal probability distribution represents the likelihood for the outcomes of a subset of variables in the joint distribution. It can be calculated by summing across certain variables in a joint distribution. For instance, using the example probability distributions for coins above, we can relate the marginal distribution for the first coin $p\left(c_{1}\right)$ to the joint distribution for both coins $p\left(c_{1},c_{2}\right)$ via (Eqn. 1)

(1) $$p\left(c_{1}\right)=\sum_{c_{2}}p\left(c_{1},c_{2}\right)$$

These ideas can be further explored by considering a system of two magically linked (dependent) coins. Suppose that each coin in isolation produces heads and tails 50% of the time like a normal, fair coin. But, when the second coin is flipped right after the first, it is more likely to take the same value as the first coin. A joint probability distribution and the associated marginal distributions for this system might look like Table 1. This system does not have a uniform probability distribution. For instance, the state $\left(c_{1}=heads,c_{2}=heads\right)$ appears 40% of the time, while the state $\left(c_{1}=tails,c_{2}=heads\right)$ appears only 10% of the time. The likelihood that variable coin 2 is heads $p\left(c_{2}=heads\right)$ is equal to the sum of $p\left(c_{1}=heads,c_{2}=heads\right)$ and $p\left(c_{1}=tails,c_{2}=heads\right)$ because those are the two possible combinations of all the coins where coin 2 is heads. Notice that all the marginal distribution values are 0.5, indicating that the coins appear in isolation to be normal, fair coins.

**Table 1.** Marginal and joint probability distributions for an example system of two dependent coins.

	$c_{1}=heads$	$c_{1}=tails$	Marginal Distributions for Coin 2
$c_{2}=heads$	$p\left(c_{1}=heads,c_{2}=heads\right)=0.4$	$p\left(c_{1}=tails,c_{2}=heads\right)=0.1$	$p\left(c_{2}=heads\right)=0.5$
$c_{2}=tails$	$p\left(c_{1}=heads,c_{2}=tails\right)=0.1$	$p\left(c_{1}=tails,c_{2}=tails\right)=0.4$	$p\left(c_{2}=tails\right)=0.5$
Marginal distributions for coin 1	$p\left(c_{1}=heads\right)=0.5$	$p\left(c_{1}=tails\right)=0.5$	

The joint distribution describe the likelihood for each possible combination of the two coins. The marginal distributions describe the likelihood for each coin alone. Marginal distributions can be found by summing across rows or columns of the joint distribution (Eqn. 1).

The conditional probability distribution is another important way to represent probabilities in systems of multiple variables. A conditional probability distribution is the likelihood of outcomes for some subset of variables given the states of other variables in a joint probability distribution. In other words, conditional probability distributions describe the likelihood to obtain outcomes of certain variables assuming that other variables are known. The conditional probability distribution (notated as $p\left(A|B\right)$ for the probability of A given B) can be related to the marginal and joint probability distributions using Bayes’ theorem (Eqn. 2):

(2) $$p\left(A|B\right)=\frac{p\left(A,B\right)}{p\left(B\right)}$$

We can use Bayes’ Theorem to calculate the conditional probability distributions (Table 2) for the example system of two dependent coins whose joint and marginal distributions are shown in Table 1. In this example $p\left(c_{1}=tails|c_{2}=heads\right)=0.2$, which means that when coin 2 is heads there is only a 20% chance that coin 1 is tails. Conversely, $p\left(c_{1}=heads|c_{2}=heads\right)=0.8$, which means that when coin 2 is heads, there is an 80% chance that coin 1 is also heads.

**Table 2.** Conditional probability distributions for the example system shown in Table 1.

Likelihood of a state of coin 1 given the state of coin 2	
$p\left(c_{1}=heads|c_{2}=heads\right)=0.8$	$p\left(c_{1}=tails|c_{2}=heads\right)=0.2$
$p\left(c_{1}=heads|c_{2}=tails\right)=0.2$	$p\left(c_{1}=tails|c_{2}=tails\right)=0.8$
Likelihood of a state of coin 2 given the state of coin 1	
$p\left(c_{2}=heads|c_{1}=heads\right)=0.8$	$p\left(c_{2}=tails|c_{1}=heads\right)=0.2$
$p\left(c_{2}=heads|c_{1}=tails\right)=0.2$	$p\left(c_{2}=tails|c_{1}=tails\right)=0.8$

The conditional probability p(A|B) describes the likelihood of a state of a given the state of B and can be related to the joint and marginal probability distributions (table 1) via Bayes’ theorem (Eqn. 2).

Throughout this tutorial, we will focus almost exclusively on discrete probability distributions for several important reasons. Primarily, when first learning about probability distributions and information, we find it is easier for most people to focus on discrete probability distributions. Working with continuous distributions involves concepts from calculus and the continuous analogs of various information theory measures can produce subtly different, but possibly confusing results (e.g., the entropy for discrete distributions is always non-negative, but the continuous analog can be negative (Cover and Thomas, 2006). In addition, continuous versions of several information theory measures discussed in this tutorial have not yet been developed. However, we wish to emphasize that information theory analyses of continuous probability distributions can be successfully employed, continuous data are best understood mathematically using continuous probability density functions, and the interested reader should consider further resources on the subject (Cover and Thomas, 2006). Of course, a great deal of neuroscience data are continuous, so we will use various techniques (e.g., binning) to convert continuous data to discrete data throughout this tutorial (see *Data Binning and Handling Continuous Data.*).

#### Converting neuroscience data to probability distributions

If casting data in terms of probability distributions is necessary to information theory analyses, how do we convert raw data from neuroscience experiments into probability distributions? Indeed, this is a nontrivial problem that has the potential to dramatically affect the outcome of information theory analyses. This process (so-called state space reconstruction) is of vital importance (Wibral et al., 2014a).

**Figure 1.**
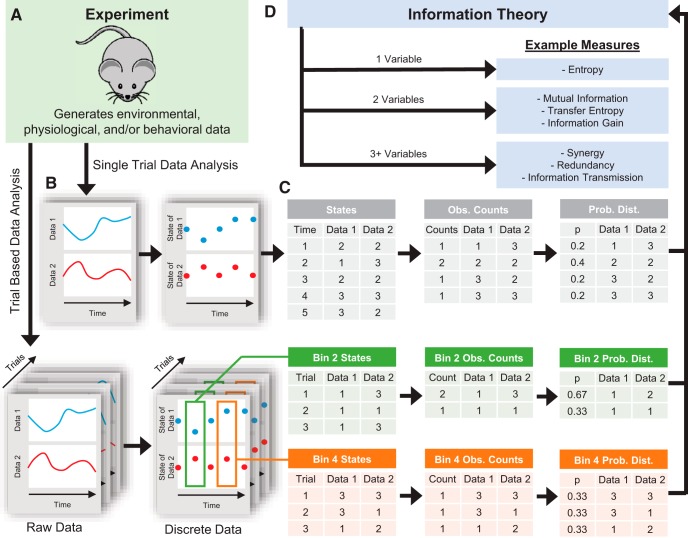
General information theory analysis protocol. ***A***, A neuroscience experiment or simulation is performed to gather environmental data (e.g., stimuli), physiologic data (e.g., voltage recordings), and/or behavioral data (e.g., animal location). ***B***, If necessary, the data are then discretized (see *Data Binning*). Some types of data (e.g., spike data) do not require discretization. In this example, two sets of data were produced, but analysis of any number of data sets is possible. ***C***, The discretized data are then converted to probability distributions by first counting the number of times each unique set of states was observed. In the case of single trial data (gray tables), the joint states for all of the data are counted to estimate the probability distribution. In the case of trial-based data (green and orange tables), the joint states are counted for all data at certain time bins across trials. ***D***, The desired information theory measure is applied to the probability distribution.

Many neuroscience experiments involve multiple observations of the system/organism under study, via either multiple trials or a single recording of a system’s behavior through time (Fig. 1*A*). The data from the experiment can then be discretized (if necessary) into states s (Fig. 1*B*, see *Data Binning* for more information about binning and see *Handling Continuous Data and Further Refinements* for alternatives to binning). (Note, we are not referring to time binning at this point, but rather binning of multiple observations of some variables across trials or time.) Admittedly, understanding this binning procedure is one of the most difficult aspects of employing an information theory analysis. Part of the problem lies in the fact that this step is highly flexible and system specific. Suppose the data consist of individual electrode voltage values through time. In this case, we could bin the values into certain voltage ranges (e.g., less than –1 mV, –1 to 1 mV, and >1 mV). (We will discuss why/how to pick certain ranges below.) Then, we could refer to all voltage measurements below –1 mV as state 1, between –1 and 1 mV as state 2, and above 1 mV as state 3.

At the most basic level, the probability of a state is estimated as the total number of observations of that state divided by the total number of observations for all states (Fig. 1*C*). For instance, if we note s as the state of the variable being recorded (e.g., electrode voltage), $N\left(s\right)$ as the number of experimental observations of state s (a so-called frequency distribution; e.g., the number of time bins where the voltage was in a certain range), and $N_{obs}$ as the total number of experimental observations, then the probability for the state s would be estimated by

(3) $$p\left(s\right)=\frac{N\left(s\right)}{N_{obs}}$$

So, if we recorded the voltage for 10,000 time bins and 1298 of those time bins produced a voltage less than –1 mV, we would find $p\left(s=1\right)=0.1298$. It should be noted that this is a form of maximum likelihood estimation (Myung, 2003), in that we assume that the data observed represent the most likely outcome of the underlying probability distribution. Once the probability distribution is estimated, the appropriate information theory measure can be applied (Fig. 1*D*, see sections below on various information theory measures).

Depending on the information measure to be used, the states could consist of a single data type (e.g., voltage on one electrode) or multiple data types (e.g., voltage recordings from multiple electrodes). If multiple data types are used, the state is then a joint state for all the variables (e.g., voltage recording 1 is less than –1 mV and voltage recording 2 is >1 mV). If the analysis utilizes single-trial data, the states could be experimentally recorded values through time, with each time bin being a unique state observation. Such an analysis will produce a time-averaged information value throughout the recording (see discussion of stationarity concerns below). If the analysis utilizes trial-based data, the states could be experimentally recorded values at a given time point across trials (e.g., 100 ms after the stimulus), with each trial being a unique state observation instead of each time bin throughout a recording being a state observation. Such an analysis will produce an instantaneous information value at a given time point in the trial (Lizier et al., 2008; Wibral et al., 2014a for other interpretations of instantaneous information values). If multiple values are being recorded through time, delays can be introduced in the time order of the states (e.g., stimulus at a given time in a trial and neural signal 200 ms later in a trial).

It is important to note that the state being observed is very flexible. For instance, with neurons the distinction between rate coding and spike timing coding has frequently been discussed (Victor and Purpura, 1996; Borst and Theunissen, 1999; Panzeri and Schultz, 2001; Van Rullen and Thorpe, 2001; Stanley, 2013). In this case, the state could be the number of spikes in a given time bin when a rate coding scheme is being investigated, or the state could be a specific pattern of spikes when a spike timing coding scheme is being investigated.

Enough observations must be performed to adequately sample the space of possible joint states. As a bare minimum, the number of observations must be greater than the number of possible joint states, though more observations are usually necessary to perform an information theory analysis. To the best of our knowledge, there are no agreed-on standards for the number of observations necessary to adequately estimate a joint probability distribution, though informal discussions with other researchers suggest >10 observations per possible state is ideal. Note that the use of significance testing via surrogate data (see *Significance Testing*) can minimize the occurrence of type 1 errors (i.e., reporting a significant information theory result when none is actually present). However, small data sets will increase the likelihood of type 2 errors (i.e., failing to report a significant information theory result when one is actually present) from surrogate data significance testing and produce bias (see *Bias in Entropy and Mutual Information*). The number of observations necessary to estimate a probability distribution has been explored to some extent in the literature (Ramos and Macau, 2017), but a great deal of attention has been paid to other methods to assess bias and estimate probability distributions (see *Handling Continuous Data and Further Refinements*).

In addition to concerns related to the number of observations, experimenters must also consider a fundamental assumption of this estimation method that each observation is produced from the same underlying probability distribution. This assumption is frequently referred to as “stationarity.” In other words, we must assume that each observation contributes to a picture of the same probability distribution (which we cannot directly access). If the underlying probability distribution is changing through our observations, the method outlined in Eqn. 3 will not produce a valid estimate of the probability distribution. Thus, the experiment must be designed in such a way that stationarity can be assumed. This can be especially important in single-trial data analysis, where the underlying probability distribution may be suspected to change throughout a recording. For instance, research should consider whether animal behavior is changing over observations (e.g., is the animal becoming satiated?) or whether neural behavior is changing (e.g., are firing rates changing?).

#### Data binning

A further complication regarding estimation of probability distributions is the fact that data from neuroscience experiments can be continuous (e.g., action potential times, voltage, calcium signal, BOLD signal, movements, positions, principle components, etc.) or discrete (e.g., action potential magnitudes, some types of stimuli, some types of behaviors, animal strain, etc.). Data that are naturally discrete, such as binary stimuli (e.g., light on versus light off), are relatively easy to use with the simple estimation method in (Eqn. 3). Other data can have discrete and nondiscrete features, such as action potentials whose magnitudes are discrete (spike versus no spike) but whose timing is continuous (though typically binned in time by the recording resolution of the experimental system). However, continuous data never precisely repeat through observations, so the number of observations for each state would trivially be 1. In other words, a particular voltage observation could be 5.3476 mV, and that voltage value will not be repeated throughout an experiment. Thus, counting the number of observations at a specific voltage value will not provide an estimate of the underlying probability distribution. (However, repeated observations can provide an estimate of the density of a continuous probability distribution; see *Handling Continuous Data and Further Refinements*.)

How should we handle continuous data? The primary solution to this problem that we will discuss in this article is to convert the continuous data to discrete data via some type of binning or discretization procedure. [See *Handling Continuous Data and Further Refinements* for a discussion of other methods for handling continuous data and Daw et al. (2003) for a general review of discretization in data analysis]. Once this binning procedure is performed, the continuous data have been made discrete, and the probability distribution estimation method described in Eqn. 3 can be applied. We will discuss two general procedures, all of which are implemented in the information theory toolbox (see *Software*). Note that it is frequently necessary to look at the data to see which discretization method will be most appropriate for the analysis and to set discretization parameters. For instance, knowledge of a neuron’s firing rate can help influence decisions about time bin size and the ranges of spike counts per bin (e.g., low-firing-rate neurons need larger time bins and finer spike count resolution, while high-firing-rate neurons need smaller time bins and less spike count resolution to adequately capture the dynamic range of the spiking behavior). In all cases, users should avoid “parameter fishing” to find binning methods that yield expected results (see *Significance Testing.*).

**Figure 2.**
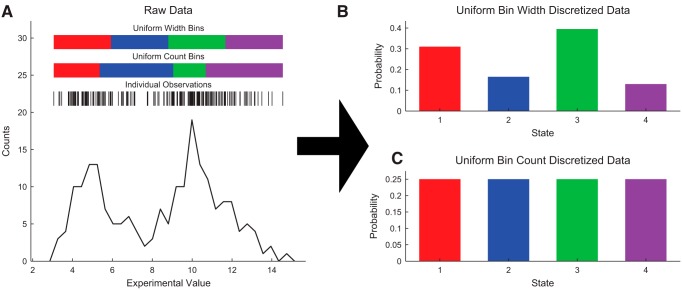
Example data discretization. ***A***, 200 example data points were randomly generated (vertical black lines represent individual data points, black plot represents a fine-resolution histogram). The data were discretized into four uniform width bins or states (top colored regions) or four uniform count bins or states (bottom colored regions). ***B***,***C***, The number of data points in each bin divided by the total number of data points was then used as the probability for each bin (state). Uniform width bins (***B***) can preserve general data distribution features (e.g., two peaks in this case), but produce some bins with low probabilities. Uniform count bins (***C***) produce uniform probability distributions, which have certain information theory advantages, but do not preserve general data distribution features.

The first binning procedure we will discuss simply involves dividing the total range of the data (i.e., minimum value observed to maximum value observed) into $N_{bins}$ number of equal-width bins (Fig. 2*A*,*B*). Note that this method preserves some large-scale patterns in the continuous distribution of the data, though that resolution is dependent on the choice of $N_{bins}$, which is a parameter of the analysis. We generally refer to this method as the “uniform width” binning method.

The second binning procedure we will discuss uses a similar method, except the data are binned into $N_{bins}$ equal-count bins (Fig. 2*A*,*C*), typically referred to herein as “uniform count” binning method. Importantly, this method does not preserve large-scale structure in continuous data distributions, but this method is especially powerful when examining relationships between variables because it maximizes the available information signal between variables (see *Entropy and Mutual Information*). Furthermore, this method can allow for the use of a single null model for the analysis of multiple variables, which can significantly decrease computation time in significance testing (see *Significance Testing.*). Finally, this method is not impacted by the range of the data or the various scales in the data. With equal width binning, an outlier data point may dramatically affect the binned structure of the data by skewing the bins. For these reasons, we used the uniform count method throughout the demonstrations presented herein, though the Neuroscience Information Theory Toolbox contains numerous demonstrations of other binning procedures (see *Software*; Extended Data).

10.1523/ENEURO.0052-18.2018.ed1 Extended Data Download Extended Data, ZIP file

Once one of these binning methods is chosen and applied to the data, the probability distribution can be estimated via the method described in Eqn. 3, where each bin corresponds to a unique state.

#### Handling continuous data and further refinements

We chose to discuss the previous discretization methods for analyses of continuous data because they are relatively simple to employ and understand, which aligns well with this article’s goal of introducing information theory analyses. However, other valuable methods of handling continuous data have been proposed previously, and other logistic concerns exist for information theory analyses. For instance, kernel-based or binless strategies exist for several information theory measures (Victor, 2002; Kraskov et al., 2004; Vicente et al., 2011). These methods use the density of the continuous data points to estimate the underlying continuous probability distribution. While these methods are very useful for some types of analyses, they are more complicated than the introductory methods discussed herein, they can involve assumptions about the data that may not hold (e.g., the data are normally distributed), and they have not been extended to recently developed multivariate information theory metrics.

The importance of time bin size and delayed interactions in various information theory analyses has been noted previously (Wibral et al., 2013, 2014a). In our discussion above, we noted that delays could be introduced between observations of variables (e.g., what is the state of the voltage on electrode 2 t seconds after the state of voltage on electrode 1?). In short, analyses of this type are prone to biases associated with assuming the incorrect interaction delay in the system (e.g., assuming t=100 ms when really t=200 ms). Therefore, it is necessary to conduct measurements at appropriate delays and/or to conduct some type of delay scanning procedure (Ito et al., 2011; Wibral et al., 2013). If there are biological reasons to select a certain delay between observations, that delay should be used. For instance, it is biologically implausible that V1 neurons will respond to visual stimuli within 1 ms. If the delay is not known, several possible delays could be scanned. For instance, the precise synaptic delay between two neurons may not be known, but delays ranging from 1 to 20 ms could be scanned to see if a certain time range corresponds to an increase in interactions.

Finally, it is important when conducting an information theory analysis to explore the dynamic range of the system. As will become clearer once we discuss the information values themselves, it is vital that the variables used actually vary across observations. Furthermore, experimenter-controlled variables (e.g., stimuli) must be varied to produce an appropriate range of responses. For instance, if the analysis receives only cases where one type of stimulus was applied, it will not be possible to observe stimulus-dependent differences in neural variables. Often it is helpful to consider trials with different types of stimuli, as well as trials without stimuli.

### Entropy

Entropy is the fundamental information theory quantity. It measures the uncertainty contained in a variable. Because information theory conceptualizes information as a reduction in uncertainty, it is necessary to quantify uncertainty before information. In other words, we must understand uncertainty before we can understand information.

The entropy $H\left(X\right)$ of a discrete random variable (call this variable X with individual states x) is (Eqn. 4) (Shannon, 1948; Cover and Thomas, 2006)

(4) $$H\left(X\right)=\sum_{x\in X}p\left(x\right){log}_{2}\left(\frac{1}{p\left(x\right)}\right)$$

Note that x∈X refers to all of the possible states x can take.

As a first step in understanding entropy, it is useful to consider the simple fair coin example from above. In that case, x could take two possible states (heads and tails), and the likelihood for both outcomes is 0.5. Therefore, if we refer to this coin as $C_{1}$, then we have (Eqn. 5):

(5) $$p\left(heads\right)=p\left(tails\right)=\frac{1}{2}$$

(6) H(C1)=∑x∈{heads,tails}p(x)log2(1p1(x))=12log2(112)+12log2(112)=log2(112)=log2(2)=1

**Figure 3.**
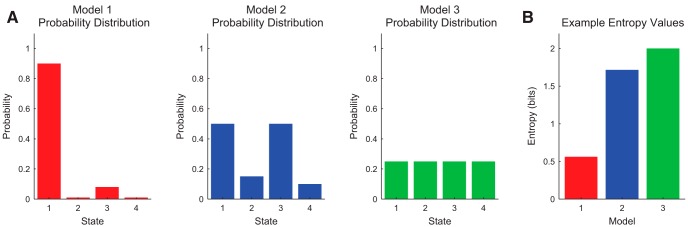
Example entropy calculations. ***A***, Example probability distributions for three models (red, blue, and green); ***B***, their associated entropy values. Model 1 was most likely to be in state 1, so it had low entropy. Model 3 was equally likely to be in all four states, so it had maximum entropy. Uniform count binning (see *Data Binning*) will produce equally likely states and maximize entropy, similar to Model 3.

Therefore, the uncertainty of a fair coin is 1 bit. Three other example systems with four possible states instead of two are shown in Fig. 3. Note that for systems with probability distributions that are more concentrated, the entropy is lower (Model 1 in Fig. 3), while for systems with evenly spread probability distributions, the entropy is higher (Model 3 in Fig. 3). If a variable is likely to be in one state (i.e., concentrated probability distribution), it has low uncertainty. Conversely, if a variable is equally likely to be in many different states (i.e., dispersed probability distribution), it has high uncertainty. This intuitively agrees with our definition of uncertainty. Note that the uniform counts binning procedure (see *Data Binning*) will produce a uniform probability distribution, which will maximize entropy (Cover and Thomas, 2006). Furthermore, a variable that is perfectly concentrated (i.e., it has only one possible state and that state has a likelihood of 1) will produce an entropy of 0. This makes sense because such a variable has no uncertainty. Finally, the entropy cannot be negative. This is because the likelihoods of the individual states cannot be larger than 1, so the argument of the logarithm cannot be <1, so the logarithm will always be positive.

As is typically done in information theory, we use logarithms with base 2 in our entropy calculations (Eqn. 4), but we could have chosen any other base. The choice of base 2 produces entropy values in units of bits. Other base values produce different units for entropy (e.g., using the natural logarithm produces units of nats). Throughout this paper, we will use units of bits because they provide two useful conceptual connections. First, computer memory is expressed in units of bytes, which are related to bits via 1 byte = 8 bits. Second, as we discussed above, we can think of bits as yes/no questions. When we conceptualize bits this way, we can think of the entropy as the average number of yes/no questions necessary to determine the state of the variable. Returning to the fair coin example, we can see that one yes/no question (e.g., “Is the coin in the heads state?”) can always allow us to determine the state of the coin, which is why the result of our entropy calculation in (Eqn. 6) yielded 1 bit of entropy.

Just as probability distributions can have more than one variable in a joint probability distribution, the joint entropy can be calculated for systems with more than one variable. The joint entropy $H\left(X,Y\right)$ of two discrete random variables (variable X with individual states x, variable Y with individual states y) is given by (Eqn. 7) (Cover and Thomas, 2006)

(7) $$H\left(X,Y\right)=\sum_{x\in X,y\in Y}p\left(x,y\right){log}_{2}\left(\frac{1}{p\left(x,y\right)}\right)$$

For the system of two independent fair coins from above (likelihood of each combination of heads and tails is 0.25), we obtain

(8) H(C1,C2)=∑x∈{heads,tails},y∈{heads,tails}p(x,y)log2(1p(x,y))=4[14log2(114)]=log2(114)=log2(4)=2

This result agrees with our interpretation of entropy as the average number of yes/no questions necessary to determine the state. We found from Eqn. 6 that we needed one question to determine the state of one coin. Because the coins are independent, we should need two yes/no questions to determine the state of two coins, which is exactly what we find in Eqn. 8. Indeed, in general, when the X and Y variable are independent (i.e., $p\left(x,y\right)=p\left(x\right)p\left(y\right)$), the joint entropy of the two variables is simply the sum of the individual entropies (recall $\sum_{x\in X}p\left(x\right)=1$) (Eqn. 9):

(9) $$H_{ind}\left(X,Y\right)=\underset{x\in X,y\in Y}{\sum }p\left(x,y\right){log}_{2}\left(\frac{1}{p\left(x,y\right)}\right)=\underset{x\in X,y\in Y}{\sum }p\left(x\right)p\left(y\right){log}_{2}\left(\frac{1}{p\left(x\right)p\left(y\right)}\right)=\underset{x\in X,y\in Y}{\sum }p\left(x\right)p\left(y\right)\left[{log}_{2}\left(\frac{1}{p\left(x\right)}\right)+{log}_{2}\left(\frac{1}{p\left(y\right)}\right)\right]=\underset{x\in X,y\in Y}{\sum }p\left(x\right)p\left(y\right)\left[{log}_{2}\left(\frac{1}{p\left(x\right)}\right)\right]+\underset{x\in X,y\in Y}{\sum }p\left(x\right)p\left(y\right)\left[{log}_{2}\left(\frac{1}{p\left(y\right)}\right)\right]=\underset{x\in X}{\sum }p\left(x\right)\left[{log}_{2}\left(\frac{1}{p\left(x\right)}\right)\right]+\underset{y\in Y}{\sum }p\left(y\right)\left[{log}_{2}\left(\frac{1}{p\left(y\right)}\right)\right]=H\left(X\right)+H\left(Y\right)$$

The final entropy quantity of interest is the conditional entropy, which quantifies the average uncertainty in a variable given the state of another variable. The conditional entropy $H\left(X|Y\right)$ of two discrete random variables (variable X with individual states x given variable Y with individual states y) is given by (Eqn. 10) (Cover and Thomas, 2006)

(10) $$H\left(X|Y\right)=\sum_{x\in X,y\in Y}p\left(x,y\right){log}_{2}\left(\frac{1}{p\left(x|y\right)}\right)$$

As an example for the joint entropy, we will again turn to the system of two independent fair coins from above. In this case, the conditional likelihood of each combination of heads and tails is 0.5, because the likelihood for the first coin to be heads is always 0.5 regardless of the state of the second coin. Thus, we obtain (Eqn. 11)

(11) H(C1|C2)=∑x∈{heads,tails},y∈{heads,tails}p2(x,y)log2(1p2(x|y))=4[14log2(112)]=log2(112)=log2(2)=1

Because the coins are independent, the conditional entropy of the first coin given the second coin should be the same as the entropy of the first coin alone, which is what we found in Eqn. 6.

As a final example of entropy, consider the system of dependent coins described in Tables 1 and 2. In this case, the joint entropy is found to be (Eqn. 12)

(12) $$H\left(C_{1},C_{2}\right)=\underset{x\in \left\{heads,tails\right\},y\in \left\{heads,tails\right\}}{\sum }p\left(x,y\right){log}_{2}\left(\frac{1}{p\left(x,y\right)}\right)=2\left[0.4*{log}_{2}\left(\frac{1}{0.4}\right)\right]+2\left[0.1*{log}_{2}\left(\frac{1}{0.1}\right)\right]=1.73$$

Because the coins are dependent, their joint entropy (1.73 bits) is less than was found for two independent coins (2 bits; Eqn. 8). In other words, for these dependent coins, there is less uncertainty about the state of the system because it is more likely to be in states where the coins match than states where they do not match. The conditional entropy for each coin in this system is identical and is found to be (Eqn. 13):

(13) $$H\left(C_{1}|C_{2}\right)=\underset{x\in \left\{heads,tails\right\},y\in \left\{heads,tails\right\}}{\sum }p\left(x,y\right){log}_{2}\left(\frac{1}{p\left(x|y\right)}\right)=2\left[0.4*{log}_{2}\left(\frac{1}{0.8}\right)\right]+2\left[0.1*{log}_{2}\left(\frac{1}{0.2}\right)\right]=0.73$$

While each coin in isolation produces heads and tails with equal likelihood and therefore has an entropy of 1 bit (Eqn. 6), the uncertainty in the state of each coin is reduced when the other is known. Therefore, the conditional entropy of each coin is <1 bit.

In all cases, not just systems with independent variables, the following intuitive relationship between entropy, joint entropy, and conditional entropy can be shown (Cover and Thomas, 2006):

(14) $$H\left(X,Y\right)=H\left(X\right)+H\left(Y|X\right)$$

In words, (Eqn. 14) says that the joint entropy of X and Y is equal to the entropy of X along, plus the remaining entropy of Y when X is known.

While the formula given in Eqn. 4 may seem unusual, it actually has several important properties that motivated its creation (Shannon, 1948; Cover and Thomas, 2006). First, $H\left(X\right)\geq 0$. In other words, the entropy can never be negative. This is very helpful because it is unclear how we would interpret negative uncertainty. Second, systems with one absolutely certain state have no entropy. This makes sense because a system that is always in one state cannot provide information, nor can another variable provide information about it. Finally, the entropy of two independent variables is simply the sum of their individual entropies (Eqn. 9) (i.e., the entropies are additive).

### Mutual information

Information theory conceptualizes information as a reduction in uncertainty in one variable when another variable is known. In other words, if learning the state of one variable reduces our uncertainty in another variable on average, then the first variable provides information about the second variable. Importantly, we are now able to quantify uncertainty using entropy, so we can quantify this reduction in uncertainty and, therefore, information. While this notion of information is conceptually similar to correlation measures like explained variance, it is important to note that the definition of information developed in information theory possesses several distinct advantages (see *Why use information theory?*, see Fig. 5 below).

Recall that the conditional entropy $H\left(X|Y\right)$ (Eqn. 10) expresses the entropy that remains in X given knowledge about Y. Thus, the total entropy of X must be equal to the entropy that remains in X after Y is learned plus the information $I\left(X;Y\right)$ provided by Y about X. Therefore,

(15) $$H\left(X\right)=H\left(X|Y\right)+I\left(X;Y\right)$$

Note that information will also be measured in bits, because the units of entropy are bits. Because entropy is always positive, it follows from Eqn. 15 that $I\left(X;Y\right)\leq H\left(X\right)$. Therefore, it is frequently advantageous in analyses of real data to discretize data using a uniform count method to maximize each variable’s entropy and thereby maximize the available information signal (see *Data Binning*).

The relatively simple expression in Eqn. 15 can be rearranged and rewritten using probability distributions (Eqns. 4 and 10) to provide an expression for information, or as it is more generally referred to in the literature, the “mutual information” (Cover and Thomas, 2006):

(16) $$I\left(X;Y\right)=H\left(X\right)-H\left(X|Y\right)=\sum_{x\in X,y\in Y}p\left(x,y\right){log}_{2}\left(\frac{p\left(x,y\right)}{p\left(x\right)p\left(y\right)}\right)$$

**Figure 4.**
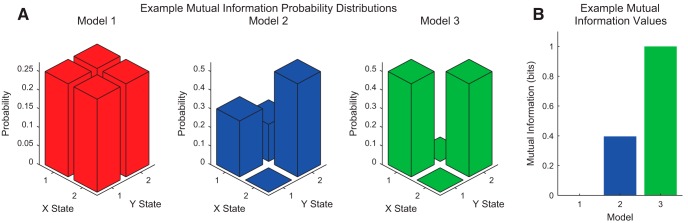
Example mutual information calculations. ***A***, Example probability distributions for three models (red, blue, and green); ***B***, their associated mutual information values. In model 1, the X and Y variables were independent, so their mutual information was zero. In model 2, knowledge of X or Y reduces our uncertainty in the state of the other variable to some extent, so nonzero mutual information was observed. In model 3, X and Y are identical, so their mutual information was maximal.

In Fig. 4, we present three example systems with their corresponding mutual information values. In model 1, knowledge of either variable does not provide information about the other variable, so the mutual information is zero. For instance, if X is known to be in state 1, Y is equally likely to be in states 1 and 2. In model 2, some information about either variable is provided by the other, so the mutual information is nonzero. In model 3, each variable perfectly predicts the other, so the mutual information is maximized at 1 bit. In this case, 1 bit is the maximum mutual information because each variable has an entropy of 1 bit.

In addition to conceptualizing information as a reduction in uncertainty, another interpretation of information is provided by the sum expression in Eqn. 16. Recall that when two variables are independent $p\left(x,y\right)=p\left(x\right)p\left(y\right)$. Thus, in Eqn. 16, for independent variables, the argument of the logarithm becomes one for all states, which produces an information of zero. This agrees with intuition, because independent variables cannot provide information about each other. In this way, the mutual information is viewed as the Kullback–Leibler distance between the true joint distribution $p\left(x,y\right)$ and the joint distribution under the assumption the data are independent p(x)p(y) (Cover and Thomas, 2006).

Note that the sum term in Eqn. 16 is symmetric in X and Y, which implies that $I\left(X;Y\right)=I\left(Y;X\right)$ (this symmetry can also be noted in an alternative expression for the mutual information: $I\left(X;Y\right)=H\left(X\right)+H\left(Y\right)-H\left(X,Y\right)$; Cover and Thomas, 2006). In other words, the information Y provides about X is equal to the information X provides about Y. For instance, in the example where I tell you the state of a flipped coin from above, my message contained information about the state of the coin, but the coin provides the same amount of information about my message. Because of this symmetry, $I\left(X;Y\right)$ from Eqn. 16 is most commonly referred to as the “mutual information.” We will employ this nomenclature throughout the remainder of this article to maintain consistency with the established literature.

**Figure 5.**
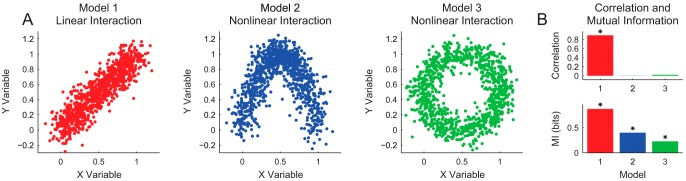
Example of linear versus nonlinear analysis methods. ***A***, Example data for three models (red, blue, and green) with linear (red) and nonlinear (blue and green) interactions; ***B***, the associated correlation coefficient and mutual information (MI) values for all three models (star: *p* < 0.05, correlation coefficient and *p*-value calculated via MATLAB, mutual information and *p*-value calculated via the Neuroscience Information Theory Toolbox; see *Data Binning and Significance Testing*, 4 bins and 1000 null data sets).

To demonstrate the differences between linear analyses and information theory using mutual information, we created three model systems: one with linear interactions and two with nonlinear interactions (Fig. 5). A linear analysis method like correlation easily detects a significant correlation coefficient among linearly related variables but does not detect a significant interaction among two data sets with nonlinear interactions. Conversely, mutual information is able to detect a significant interaction in all three cases. While this example clearly demonstrates information theory’s ability to detect nonlinear interactions, it is important to note that the resulting mutual information values do not produce a model that describes the relationship between the variables (see *What can information theory tell you? and Model Building*).

Now that we have an expression for mutual information between two variables, it is natural to expand to systems of three or more variables. The most straightforward method for measuring the information between three variables is to use mutual information between two variables, but make one of the variables a joint (or “vector valued”) variable of two variables. When two variables are combined in this way, we consider each unique combination of states for the two joined variables as a unique state for the joint set. For instance, a joint variable consisting of two coins has four states: each possible combination of heads and tails. The mutual information between a joint variable constructed from two X variables ($X_{1}$ and $X_{2}$) and another variable Y can be expressed using (Eqn. 17)

(17) $$I\left(\left\{X_{1},X_{2}\right\};Y\right)=\sum_{x_{1}\in X_{1},x_{2}\in X_{2},y\in y}p\left(x_{1},x_{2},y\right){log}_{2}\left(\frac{p\left(x_{1},x_{2},y\right)}{p\left(x_{1},x_{2}\right)p\left(y\right)}\right)$$

Using this method, we can calculate the mutual information between one variable and two other variables considered together. This type of analysis can be very helpful in neuroscience applications. For instance, we might ask how much information two neurons provide about a stimulus or behavior together instead of individually. Below, we will explore other methods for quantifying the information between more than two variables.

Mutual information can be further expanded by considering the mutual information between two variables conditioned on a third variable. This measure is referred to as conditional mutual information and is given by (Eqn. 18)

(18) $$I\left(X;Y|Z\right)=H\left(X|Z\right)-H\left(X|Y,Z\right)=\underset{x\in X,y\in Y,z\in Z}{\sum }p\left(x,y,z\right){log}_{2}\left(\frac{p\left(x,y|z\right)}{p\left(x|z\right)p\left(y|z\right)}\right)$$

The conditional mutual information allows us to examine interactions between two variables while taking into account the effects of a third variable. For instance, suppose we had three magic coins that always produced identical results. In this case, the mutual information between any pair of coins would be one bit. However, the conditional mutual information between any pair given the third coin would be zero bits. This is because the information each coin provides about the other can be explained or provided by the third coin. Another example illustrates that the inclusion of a third variable can increase the information between two variables. Suppose we had two independent, unbiased coins and a third magic coin that always produced a heads when either the first coin or the second coin produced a heads and tails only when both coins produced tails (i.e., an OR operation). In that case, the mutual information between the first two coins would be 0, but the conditional mutual information between them conditioned on the third coin would be 0.19 bits. Thus, the third coin creates conditional dependence between the otherwise independent coins. Conditional mutual information will be especially helpful when examining causal relations.

### Transfer entropy

As we discussed above, the probability distributions used in information theory calculations can be produced from many different types of data, possibly with different temporal relations. When a certain temporal ordering is used with conditional mutual information, an information theory measure called “transfer entropy” is produced. Transfer entropy is given by (Schreiber, 2000)

(19) $$TE\left(X\to Y\right)=I\left(Y_{future};X_{past}|Y_{past}\right)=H\left(Y_{future}|Y_{past}\right)-H\left(Y_{future}|X_{past},Y_{past}\right)=\underset{y_{f}\in Y_{future},x_{p}\in X_{past},y_{p}\in Y_{past}}{\sum }p\left(y_{f},x_{p},z\right){log}_{2}\left(\frac{p\left(y_{f},x_{p}|y_{p}\right)}{p\left(y_{f}|y_{p}\right)p\left(x_{p}|y_{p}\right)}\right)$$

Transfer entropy measures the information about the future state of a variable ($Y_{future}$) provided by another variable in the past ($X_{past}$) given the information provided by the past state of the variable ($Y_{past}$). Once these temporal relationships are defined, transfer entropy can be interpreted as a better measure of causal influence from X to Y than merely the mutual information between $X_{past}$ and $Y_{future}$, because transfer entropy measures the changes caused in Y from X that are not accounted for by the history of Y alone. However, it is possible for no causal interaction to exist from $X_{past}$ to $Y_{future}$, but yet to still observe a nonzero transfer entropy result. For instance, even when considering $Y_{past}$, it is possible that some third unmeasured variable controls $X_{past}$ and $Y_{future}$, rendering the interaction from $X_{past}$ to $Y_{future}$ noncausal.

As an example of transfer entropy, consider simultaneously measuring the firing rate through time of two neurons, X and Y. Assume that X sends an inhibitory connection to Y such that when X fires, Y stops firing. Knowing the past state of X allows you to predict the future of Y better than predicting the future of Y with only the past of Y. This type of interaction would result in increased transfer entropy from X to Y.

Fundamentally, transfer entropy is simply conditional mutual information with certain assumptions about temporal order and variable source, which allows it to serve as a measure of causal influence. Due to the widespread interest in neural connectivity (Bullmore and Sporns, 2009; Friston, 2011), transfer entropy has been widely used in the literature (for example, Honey et al., 2007; Lizier et al., 2008; Ito et al., 2011; Vicente et al., 2011; Timme et al., 2014b, 2016; Wibral et al., 2014b; Nigam et al., 2016; Bossomaier et al., 2016). Numerous methods have been employed to define past and future state (Staniek and Lehnertz, 2008; Ito et al., 2011; Wibral et al., 2013; Timme et al., 2014b). These methods allow for the exploration of interactions over certain time scales, the search for interactions with set delays (e.g., synaptic connectivity), or interactions involving patterns of activity. An interesting alternative measure called the directed information has also been used in the literature (Quinn et al., 2011).

**Figure 6.**
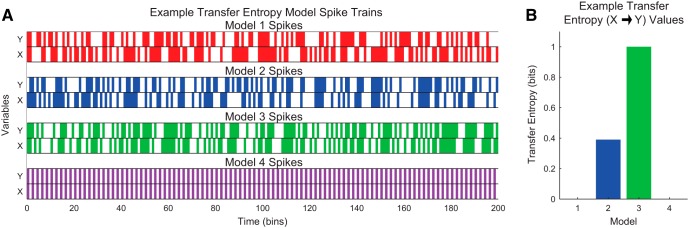
Example transfer entropy calculations. ***A***, Example model spike trains (color bands: spikes); ***B***, their associated transfer entropy values. Model 1 contained independent neurons, so it produced zero transfer entropy. Models 2 and 3 contained interactions from neuron X to Y. In model 3, neuron X’s state precisely determined neuron Y’s state one time step in the future, which produced maximal transfer entropy. In model 4, neuron X’s state precisely determines neuron Y’s state, but the past of neuron Y also determines its future, so it produced zero transfer entropy.

In Fig. 6, we present four example single-trial systems (neuron spike trains) with example data through time and their resulting transfer entropy values. For the sake of simplicity in this introduction, we will assume the past state occurs one time bin before the future state. Models 1 through 3 possessed increasing interaction strength between the state of X at a given time with the state of Y one time step later. As expected, these models produced increasing transfer entropy values. Model 4 possessed a strong interaction between the state of X at a given time with the state of Y one time step later, but that interaction could be explained with the past state of Y, resulting in zero transfer entropy. This demonstrates the conditional aspect of transfer entropy.

### Partial information decomposition

As we mentioned above, we can move beyond pairs of variables to consider information between groups of variables. The simplest such extension is to consider the mutual information $I\left(\left\{X_{1},X_{2}\right\};Y\right)$ between two variables taken together ($X_{1}$ and $X_{2}$) and a third variable Y via Eqn. 17 (as a more advanced alternative, see (Pica et al. (2017)) for recent work on treating all variables equally). While this step alone is helpful in many circumstances, it is possible to decompose this mutual information into several other useful and intuitive components. For instance, we might ask what portion of $I\left(\left\{X_{1},X_{2}\right\};Y\right)$ is provided redundantly by both $X_{1}$ and $X_{2}$ (what information overlap about Y exists between $X_{1}$ and $X_{2}$), or uniquely by $X_{1}$ or $X_{2}$ alone (what information does $X_{1}$ provide about Y that $X_{2}$ does not and vice versa), or synergistically by both $X_{1}$ and $X_{2}$ together (what bonus information do $X_{1}$ and $X_{2}$ provide about Y when both are known simultaneously). This dissection is generally referred to as the partial information decomposition (Williams and Beer, 2010). Quantifying these relationships can provide a great deal of insight into how a system functions. If all of the information in $I\left(\left\{X_{1},X_{2}\right\};Y\right)$ is provided redundantly, then we know $X_{1}$ and $X_{2}$ are doing the same thing, at least in an information theoretic sense. If all of the information in $I\left(\left\{X_{1},X_{2}\right\};Y\right)$ is provided uniquely by $X_{1}$, then we know $X_{2}$ is not providing information about Y. If all of the information in $I\left(\left\{X_{1},X_{2}\right\};Y\right)$ is provided synergistically, then we know $X_{1}$ and $X_{2}$ are somehow working together or engaged in some type of complex interaction with Y.

Note that we have not provided any explicit mathematical definition for the synergy, redundancy, and unique information concepts that we are invoking. Rather, we are relying on intuition regarding what “synergy” means in the context of information. The following equations express the intuitive relationships between the relevant mutual information terms and the synergy S, redundancy R, and unique information U1 and U2 components:

(20) $$I\left(\left\{X_{1},X_{2}\right\};Y\right)=S\left(X_{1},X_{2};Y\right)+R\left(X_{1},X_{2};Y\right)+U1\left(X_{1},X_{2};Y\right)+U2\left(X_{1},X_{2};Y\right)$$

(21) $$I\left(X_{1};Y\right)=R\left(X_{1},X_{2};Y\right)+U1\left(X_{1},X_{2};Y\right)$$

(22) $$I\left(X_{2};Y\right)=R\left(X_{1},X_{2};Y\right)+U2\left(X_{1},X_{2};Y\right)$$

In (Eqn. 20), we take the total information provided by $X_{1}$ and $X_{2}$ together to be equal to the four underlying components (synergy, redundancy, and unique information terms). In Eqn. 21, we take the information provided by just $X_{1}$ to be equal to the redundancy and the unique information provided by $X_{1}$. We include only these two terms because the unique information from $X_{2}$ is not provided by $X_{1}$ and because the synergy is not provided by $X_{1}$ alone.

While the mutual information expressions in Eqns. 20, 21, 22 can be calculated easily using Eqns. 16–17), it is not possible to derive mathematical expressions for the synergy, redundancy, and unique information without further measures. However, notice that if we had access to a measure for redundancy or unique information, the other components could be found easily via basic algebra.

Several researchers have put forward candidate measures for redundancy or unique information to address this problem (Williams and Beer, 2010; Harder et al., 2013; Bertschinger et al., 2014; Griffith et al., 2014; Ince, 2017; Finn and Lizier, 2018; see Quax et al., 2017 for an alternative information theory approach to measuring synergy). In fact, this area of information theory is currently undergoing rapid development, with numerous previous methods for analyzing synergy and redundancy in neuroscience contexts (Brenner et al., 2000; Schneidman et al., 2003a,b) being improved on. We feel strongly that it is beyond the scope of this introductory tutorial to thoroughly review all of these newly introduced measures for redundancy and unique information. Doing so properly would require an entire article. However, we also feel strongly that some of these new methods should be included in this tutorial because they represent new powerful tools for the analyses of neural data that will be of interest to readers and because related concepts have been used in neuroscience for many years.

In an attempt to thread this pedagogical needle, we have decided to focus on the first measure introduced: the minimum information (Williams and Beer, 2010). The minimum information possesses several advantages over other candidate measures in that it is relatively straightforward, it can be expanded to any number of X variables (in principle, though see Ince (2017) as an exception), and it does not require numerical approximation, which is especially relevant when analyzing large amounts of data. As described by Williams and Beer (2010), the minimum information ($I_{min}$) can be interpreted as a measure of redundancy. It is given by (Eqn. 23)

(23) $$I_{min}\left(X_{1},X_{2};Y\right)=\sum_{y\in Y}{min}_{X_{i}}\left[\sum_{x_{i}\in X_{i}}p\left(x_{i},y\right){log}_{2}\left(\frac{p\left(x_{i},y\right)}{p\left(x_{i}\right)p\left(y\right)}\right)\right]$$

Note that the minimum information is very similar to the mutual information as expressed in Eqn. 16, except that there is a minimum operation over $X_{1}$ and $X_{2}$. Thus, the minimum information measures the smallest overlap in information provided by both $X_{1}$ and $X_{2}$ about each state of Y individually. We then interpret this smallest overlap as the redundancy portion because it is the amount of information provided by both $X_{1}$ and $X_{2}$ individually. For instance, if $X_{1}$ provided 0.2 bits of information about a state of Y, but $X_{2}$ provided 0.3 bits for that state of Y, then only 0.2 bits of information would be redundantly provided by both $X_{1}$ and $X_{2}$ individually. Once we equate the minimum information with the redundancy (i.e., set $R\left(X_{1},X_{2};Y\right)=I_{min}\left(X_{1},X_{2};Y\right)$), the remainder of the decomposition terms can be found easily via (Eqns. 20, 21, 22).

**Figure 7.**
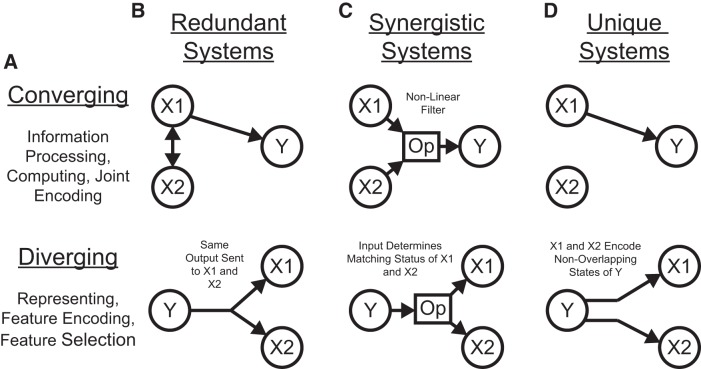
Partial information interpretations and example systems. ***A***, Though the partial information decomposition does not require explicit time ordering, it is frequently helpful to apply converging or diverging ordering to the interactions. ***B***, Example of purely redundant systems. The X variables provided the same amount of information about each state of Y. ***C***, Example purely synergistic systems. The X variables alone provided no information about Y, though they did together via a nonlinear operation (Op). ***D***, Example purely unique systems. In the converging example, only $X_{1}$ provided information about Y. In the diverging example, each X variable provided information about different states of Y. The joint probability distributions for these systems are listed as extended data in Fig. 7-1.

Our discussion of the partial information decomposition has so far ignored time ordering. However, it is frequently useful in neuroscience applications to consider a time-ordered structure where information moves from $X_{1}$ and $X_{2}$ to Y (converging) or from Y to $X_{1}$ and $X_{2}$ (diverging) (Fig. 7*A*). In the converging case, we can think of Y as processing information, computing, or jointly encoding information from $X_{1}$ and $X_{2}$. This manner of conceptualizing the partial information decomposition could be useful in experiments where two types of stimuli are presented to an animal simultaneously while a neural variable is recorded, or when two presynaptic neurons are simultaneously recorded with a shared postsynaptic target neuron. This converging framework has also been combined with transfer entropy to include the history of the receiver variable (Williams and Beer, 2011). In the diverging case, we can think of $X_{1}$ and $X_{2}$ as representing or encoding different features of Y. A common neuroscience experiment that could use this framework would include a stimulus presented to an animal while simultaneously recording two neural variables.

10.1523/ENEURO.0052-18.2018.f7-1 Extended Data Figure 7-1 Download Figure 7-1, DOCX file

While we intuitively motivated the definitions of redundancy, synergy, and unique information above, it is often very helpful to consider specific examples (see below for several neuroscience-specific examples of redundancy, synergy, and unique information). For redundant systems, we present converging and diverging examples in Fig. 7*B*. In the converging case, $X_{1}$ and $X_{2}$ behave identically in both cases, and the states of $X_{1}$ and $X_{2}$ precisely determine the state of Y. In the diverging case, the same output from Y is always sent to both $X_{1}$ and $X_{2}$. In both of these cases, $X_{1}$ and $X_{2}$ each provide information about Y, but it is the same amount of information about the same states of Y, so the information is redundant.

Example synergistic systems are shown in Fig. 7*C*. In the converging case, Y performs a nonlinear operation (Op) on $X_{1}$ and $X_{2}$ such that when $X_{1}$ and $X_{2}$ match, Y is one state, but when $X_{1}$ and $X_{2}$ do not match, Y is in another state. In the diverging case, the state of Y determines if $X_{1}$ and $X_{2}$ will be in the same state. In both of these cases, it is necessary to know the states of $X_{1}$ and $X_{2}$ together to gain information about Y. Neither $X_{1}$ nor $X_{2}$ provide information about Y alone, but together they do. Thus, the information must result from $X_{1}$ and $X_{2}$ working together, which makes the interactions synergistic.

Finally, example systems with unique information are shown in Fig. 7*D*. In the converging case, only $X_{1}$ determines the state of Y. In fact, $X_{2}$ provides no information about Y. In the diverging case, $X_{1}$ and $X_{2}$ encode non-overlapping states of Y. Therefore, the information from $X_{1}$ and $X_{2}$ each is unique, although they both provide the same amount of information individually.

We wish to emphasize that our use of the minimum information is not a declaration that it is the superior measure. While the minimum information possesses distinct advantages over other methods, it has been shown that it measures the redundant amount of information as opposed to the redundant content, which several authors find counterintuitive (Harder et al., 2013; Bertschinger et al., 2014; Griffith et al., 2014; Ince, 2017). For instance, consider an example system with the joint probability distribution given by Table 3. This system produces a redundancy of 1 bit and a synergy of 1 bit when analyzed with $I_{min}$. However, the $X_{1}$ and $X_{2}$ variables provide information about different combinations of states of Y. $X_{1}$ differentiates between Y=0,2 and Y=1,3, while $X_{2}$ differentiates between Y=0,1 and Y=2,3. Thus, we can see that $X_{1}$ and $X_{2}$ provide the same amount of information (1 bit) about the same individual states of Y, so $I_{min}$ finds a redundancy of 1 bit, despite the difference in the content in the information. We feel this is an important feature of $I_{min}$ that readers should keep in mind, not an indication that $I_{min}$ produces invalid results. If you find this interpretation lacking, we recommend you explore the other methods currently available that seek to improve on $I_{min}$ (Harder et al., 2013; Bertschinger et al., 2014; Griffith et al., 2014; Ince, 2017; Finn and Lizier, 2018).

**Table 3.** Joint probability distribution for a system that demonstrates redundancy is a measure of information quantity, not content.

*X*_1_	*X*_2_	*Y*	*p*(*x*_1_,*x*_2_,*y*
0	0	0	0.25
1	0	1	0.25
0	1	2	0.25
1	1	3	0.25

In addition to the three-variable formulation discussed above, the partial information decomposition can be expanded to include additional X variables. The mathematics involved in this expansion is somewhat complex (Williams and Beer, 2010), so we will forgo discussing it here. The partial information decomposition has also been adapted to transfer entropy measures between three variables (Williams and Beer, 2011), as well as measures of information transfer and information gain (Beer and Williams, 2014). Information transfer is conceptually similar to transfer entropy, where information is moving from some past state to a future state, except that information transfer measures information moving from one variable to another variable about a third variable. Information gain measures the information a variable gains in time about another variable. The information gained by Y through time about X is given by (Eqn. 24)

(24) $$I_{G}\left(X;Y_{t}\right)=I\left(X;Y_{future}\right)-I_{min}\left(Y_{future},Y_{past};X\right)$$

The information transferred from Y to Z about X is given by (Eqn. 25)

(25) $$I_{T}\left(X;Y_{past}\to Z_{future}\right)=I_{min}\left(Z_{future},\left\{Z_{past},Y_{past}\right\};X\right)-I_{min}\left(Z_{future},Z_{past};X\right)$$

Note that the precise delays and time bin structure for past and future states must be selected, similar to transfer entropy. Because these measures represent more advanced techniques, we will provide only the expressions here and direct the interested reader to further examples in the literature (Beer and Williams, 2014). An alternative method for measuring information transmission about some other variable can be found in Ince et al. (2015).

### Bias in entropy and mutual information

Several researchers have previously discussed biases associated with different methods for estimating information values from continuous data and/or small data sets (Treves and Panzeri, 1995; Wolpert and Wolf, 1995; Panzeri and Treves, 1996; Paninski, 2003; Nemenman et al., 2004; Panzeri et al., 2007; Bonachela et al., 2008), noting that limited data tend to bias information results. Below we will discuss several software packages that use various bias-correction algorithms to address this problem. Given the introductory nature of this article and the fact that these bias-correction techniques have not been developed for all of the information theory measures we will discuss, we will not employ these techniques in this tutorial. However, we feel it is important to discuss how bias can affect an analysis.

In simple cases, these biases can be understood as an interplay between the inherent noise associated with any analysis using limited amounts of data and the fact that information theory metrics must be greater than or equal to zero. Thus, noise in the estimate of the probability distribution produces a nonzero information result, even if the true underlying probability distribution would produce an information result of zero. Frequently, the most straightforward means of accounting for this bias when comparisons are performed between two information theory values is via significance testing (see *Signficant testing*). However, it is important to note the possible presence of bias when quoting information values. In other words, a measurement of 0.2 bits of entropy for the spiking activity of a neuron may be highly relevant within an analysis, but it is important to note that the true entropy of the spiking activity of the neuron may not be 0.2 bits, depending on the number of data points used in the calculation and the analysis techniques employed (e.g., number of bins).

**Figure 8.**
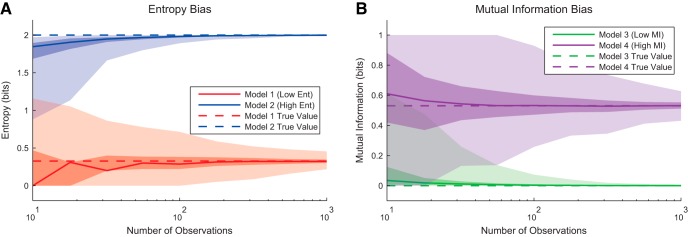
Example bias in entropy and mutual information calculations. ***A***, Distributions of entropy values for low (0.33 bits) and high (2 bits) models as a function of number of observations. Entropy values tended to be biased downwards, though some trials produced elevated entropy values for trials with few observations. The probability distribution models were $p_{low}=\left\{0.95,0.04,0.009,0.001\right\}$ and $p_{high}=\left\{0.25,0.25,0.25,0.25\right\}$. The binning method (four total bins) allowed for a maximum entropy of 2 bits. ***B***, Distributions of mutual information values for low (0 bits) and high (0.53 bits) models as a function of number of observations. Mutual information values tended to be biased upwards, though some trials produced lower mutual information values for trials with few observations. Both models had two variables, each with two states. In the low-mutual-information model, all joint states were equally likely (i.e., independent variables). In the high-entropy model, the matching joint states had a probability of 0.45 and the other joint states had a probability of 0.05. The binning method (four total joint states) allowed for a maximum mutual information of 1 bit. Dark fringe represents interquartile range, and light fringe represents extremum range over 1000 trial simulations for each model and each unique number of observations.

As examples of the effects of bias associated with small amounts of data in entropy and mutual information calculations, we produced several simple model systems. First, we produced models of low (0.33 bits) and high (2 bits) entropy. We randomly selected observations from these probability distributions, estimated the probability distributions using these observations and the methods described above, and calculated the entropy. In Fig. 8*A* we can see that as more observations were performed, the estimated entropy values approached the entropy values for the true probability distributions. For few observations, the estimated values were biased downward, though several individual trials produced elevated entropy values for the low entropy model. We performed a similar simulation with simple low (0 bits) and high (0.53 bits) mutual information models (Fig. 8*B*). Again, when few observations were performed, the estimated values varied widely, with a bias toward higher mutual information values.

### Significance testing

So far, we have discussed the logistics of converting neuroscience data to probability distributions and numerous information theory measures that can be applied to probability distributions to gain useful insights into interactions in a system. The results of such analyses will always be numbers greater than or equal to zero. Importantly, a real experimental system will rarely produce an information theory measurement of precisely zero even when no interactions actually exist between the variables because of the presence of noise. In addition, bias can dramatically alter the results of information theory analyses (see *Bias in entropy and mutual information*). Therefore, a vital step of any information theory analysis is to assess which information theory measurements are significant.

Surrogate data testing or Monte Carlo analysis is frequently the solution to significance testing in information theory analyses (Lindner et al., 2011; Timme et al., 2014b; Wibral et al., 2014a; Asaad et al., 2017). This type of analysis is performed by generating surrogate null model data that preserve certain aspects of the data while randomizing other aspects. Once the information theory analysis is applied to the surrogate data, a distribution of null model information theory values can be compared to the information theory value from the real data. The proportion of null model information theory values that are found to be larger than or equal to the real data are then taken as an estimate of the *p*-value for the information theory result from the real data.

The randomization procedures necessary to generate surrogate null model data can be performed before creating probability distributions (e.g., spike jittering (Rolston et al., 2007; Timme et al., 2014b)) or after creating probability distributions. Randomization before converting to probability distributions is highly system specific. Thus, we will primarily focus on randomization of the probability distribution, because those techniques can be generally applied to any type of data. Furthermore, if the equal counts method of data binning is used (see *Data Binning*), the same null model data can be applied to different variables in many circumstances, greatly improving computational efficiency. This is possible with equal counts binning because the marginal distributions are identical across different variables. The marginal distributions have been rendered uniform via the equal counts binning method to maximize entropy. Typically, preserving these marginal distributions is the only constraint on the randomization method (see below). Still, in any case, it is important to randomize conservatively. For instance, in the case of spike train jittering, it is frequently best to jitter by small amounts to retain large-scale changes in firing rate caused by bursts. Furthermore, if transfer entropy is to be applied to the data, it is often best to not jitter the receiver neuron spike train to preserve the autocorrelation between the past and future states of Y (Eqn. 19).

Null model data can be created after generating probability distributions by randomizing the number of observations in joint states while preserving the number of observations for each state of each variable (i.e., by preserving the marginal distributions). For instance, suppose we have two magic coins that always produce the same flip result. Furthermore, suppose we flip each coin 10 times and produce 5 heads and 5 tails for each coin. The true observations from these data, as well as the observations from a randomly chosen null model where the joint observations are randomized while preserving the number of observation for each coin alone, are shown in Table 4.

**Table 4.** True and null surrogate observations for a hypothetical experiment involving 10 flips of two magically linked coins.

	Real data		Null surrogate data	
	C_2_ = H	C_2_ = T	C_2_ = H	C_2_ = T
C_1_ = H	5	0	3	2
C_1_ = T	0	5	2	3

The null model observations in Table 4 can be converted to a probability distribution via Eqn. 3, and any information measure that can be applied to the real data can be applied to the null model data. This process can be repeated many times to generate a null distribution of information values, which allows for an estimate of the *p*-value for the real data via the process described above.

To demonstrate the significance testing process using randomized probability distributions, we will use a simple model system with two variables (X and Y) and a measurement of their mutual information. Each variable can take one of two possible states. We varied the interactions in the model using a parameter a such that (Eqn. 26)

(26) $$p\left(x=1,y=1\right)=0.25\left(1+a\right)$$

$$p\left(x=1,y=2\right)=0.25\left(1-a\right)$$

$$p\left(x=2,y=1\right)=0.25\left(1-a\right)$$

$$p\left(x=2,y=2\right)=0.25\left(1+a\right)$$

Thus, when a=0, there was no relationship between X and Y, and their mutual information should be zero in a perfect system. When a=1, X and Y were copies and their mutual information should be one in a perfect system. However, in real experiments, it is necessary to estimate the probably distribution from observations (i.e., Eqn. 3), so the estimated distribution will not be identical to the real probability distribution, which will most likely produce nonzero mutual information results even when a=0.

**Figure 9.**
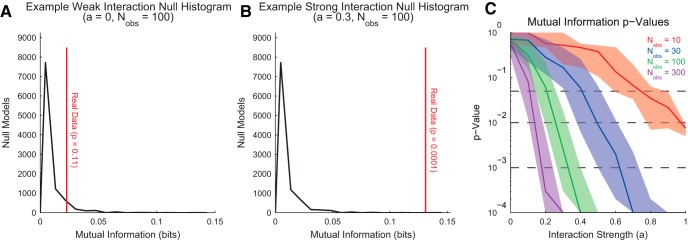
Example significance testing for mutual information via surrogate data null models. ***A***,***B***, Example histogram of null model (randomized real data) mutual information values and the mutual information value from the real data (red line) for a system with no interactions (***A***) and for a system with interactions (***B***). As expected, the *p*-value in ***A*** indicates that the null model (X and Y are independent) cannot not be rejected. In ***B***, the *p*-value is low enough to reject the null model. ***C***, *p*-values for models with different numbers of observations as a function of interaction strength (100 models generated for each a value and number of observations, solid line: median, fringe: interquartile range). Larger interaction strengths produced lower *p*-values, and models with more observations could detect weaker interactions. The minimum *p*-value resolution available in this demonstration was 0.0001 because 10,000 surrogate data sets were generated for each real data set.

We first considered an example hypothetical experiment that conducted 100 observations from a model with no interactions between X and Y (Fig. 9*A*). Despite the fact that the true underlying probability distribution governing this experiment had no mutual information, a nonzero mutual information result was observed due to random fluctuations in the observations. However, when the data were randomized 10,000 times, the distribution of null information values enveloped the value from the real data. Thus, a high *p*-value estimate was produced, and this result would not typically be recognized as significant.

Next, we considered an example hypothetical experiment that also had 100 observations, but the model contained interactions between X and Y (Fig. 9*B*). A nonzero mutual information result was observed, but the distribution of mutual information values from the null model were almost exclusively less than the mutual information result produced by the real data. Thus, a low *p*-value estimate was produced and this result would typically be recognized as significant.

Finally, we examined models with different interaction strengths and number of observations (Fig. 9*C*). As expected, when more observations were performed, weaker interactions could be detected as significant.

Selecting the number of surrogate data sets to generate is an important concern with this method of *p*-value estimation. In the examples shown above, we used 10,000 surrogate data sets, which allowed for a *p*-value resolution of 0.0001 (i.e., 1/10,000). Note that using this method, if all of the surrogate data sets produce information values less than the real value, it is only possible to estimate the *p*-value as $p<1/N_{rand}$ [or, to provide a fixed value, $p=1/\left({2N}_{rand}\right)$], not p=0. In addition to improved resolution, additional surrogate data sets may be helpful when performing multiple comparison corrections for many information values (e.g., stimulus encoding by many neurons simultaneously) by lowering the minimum *p*-value. Therefore, it is advantageous to use as many surrogate data sets as possible. However, researchers must weigh the desired or required *p*-value resolution against available computational resources when determining the right number of surrogate data sets to use in their analyses.

Frequently the question arises of how to judge the magnitude of an information theory result. If an analysis yields a result of 0.5 bits, is that a lot? The answer to this question is highly analysis specific, which prevents interpreting these values in the absence of additional testing. If the data are discretized as discussed in *Data Binning*, the maximum allowed information result will be related to the number of bins used, with more bins yielding higher maximum information values. Frequently, a helpful method for judging the magnitude of an information theory result other than entropy is to compare the value to the entropy. For instance, if the mutual information between two variables is 0.5 bits, but each of the variables has an entropy of 0.5 bits, then the variables completely predict each other. In a different case where each variable has an entropy of 5 bits, a mutual information of 0.5 bits indicates less predictive ability.

Because the information theory analyses performed herein serve as demonstrations, we will forgo significance testing in the demonstrations. That said, we wish to emphasize that significance testing is necessary to draw meaningful conclusions from real data. To aid in this process, the software package included with this article is capable of conducting significance testing as discussed above (see *Software*).

In addition to randomizing the supplied data on a measurement-by-measurement basis, the software also allows for the user to supply a previously calculated null model. Using a previously calculated null model has the potential to yield better *p*-value resolution by running one null model with many surrogate data sets and greatly reduce calculation by removing the need to analyze surrogate data for each measurement. However, this tactic will yield these advantages only if the same null model can be applied to numerous measurements, which requires the same marginal distributions across all the measurements. Using uniform counts binning will frequently produce the same marginal distributions for the underlying variables, making this process possible. For instance, if all variables in an analysis possess the same number of observations and uniform counts binning is used, then all variables will have the same uniform marginal distributions. (Note that care must be taken to test that the marginal distributions are identical in the event of observations with identical values.)

Finally, we must acknowledge two other important issues surrounding significance testing. First, it is vital to consider the number of significance tests performed and to control for multiple comparisons (e.g., via Bonferroni correction or false discovery rate control; Benjamini and Hochberg, 1995; Benjamini and Yekutieli, 2001). This is especially relevant when setting analysis parameters such as number of bins, bin size, and delays. It is not appropriate to go parameter fishing to find a bin size that produces significant results. Parameters should be set initially based on the amount of available data and prior knowledge of the system so the number of significance tests can be reduced. However, in any case, multiple comparison corrections must be performed.

Second, the entire paradigm of null hypothesis significance testing is itself a topic of some controversy (McShane et al., 2017). To a large extent, we agree that an over emphasis on *p*-values without descriptions of effect sizes, effect uncertainty, or models that explain the data are not helpful to the advancement of science. A thorough discussion of this topic is beyond the scope of this tutorial, but we wish to emphasize that simply quoting a significant result in an information theory analysis (e.g., animal strain 1 showed significant mutual information, but animal strain 2 did not) is less than ideal. It is important to note that information theory allows researchers to move beyond simply quoting *p*-values because information theory analyses produce results in bits, which allows for a direct measurement of effect size (though bias effects must be considered as well; see *Bias in Entropy and Mutual Information*). In other words, a difference in mutual information results of 1 bit indicates a smaller effect size than a difference of 2 bits. The ability to measure effect sizes and perform significance testing in a model-free manner makes information theory a valuable tool, but, especially in the context of debates about null hypothesis significance testing, it is not the only tool that should be used (see *Model Building*).

### Model building

We feel it is important to discuss the place of an information theory analysis in neuroscientific studies. As we discussed above (see *What can information theory tell you?*), information theory is very helpful for detecting complex interactions between variables, but it is not capable of providing a model that explains the gathered data. In other words, information theory can provide insights about which variables are related and how they are related (e.g., neuron 1 influences neuron 2, but not vice versa), but it cannot provide a unique model that explains those interactions (e.g., neuron 1 makes an excitatory synapse on neuron 2). Indeed, it is possible for systems governed by different rules to produce identical information theory results (James and Crutchfield, 2017). Of course, the model-free nature of information theory makes it an extremely powerful tool because it requires no assumptions about how the variables are related (though it can require some assumptions related to data analysis such as binning, see *Discussion*). This is especially relevant in complex systems where researchers frequently do not know what model should explain the data or do not want to restrict their analyses to certain types of models. However, the creation of these models is one of the most significant goals of science. In general, we seek to create compact, simplified models that explain vast oceans of data in every field of science. Therefore, to completely describe the place of information theory analyses in scientific research, it is necessary to emphasize the crucial step of model building as the step following information theory analyses. Information theory is only capable of eliminating some possible explanations for data, not identifying the one true explanation. Unlike the information theory analyses discussed above, model building is highly system dependent. Furthermore, we are aware of no studies that both perform an information theory analysis and then build a model using the results of that analysis for guidance. Thus, in this article it will be possible only to provide general suggestions about model building techniques to use following an information theory analysis.

A first general step to guide model building intuition is to examine the probability distribution to which the information theory analysis was applied. If, for instance, the mutual information was calculated between the firing rate of a neuron and a stimulus, the probability distribution can provide information about whether the neuron became more active when the stimulus was on or if it became less active. If, for instance, the transfer entropy was calculated between the firing rates of two neurons, the probability distribution can often determine if the relationship is excitatory or inhibitory. Unfortunately, simply examining the probability distribution can reveal complex nonlinear relationships, especially when more than two variables are involved. Furthermore, automated methods to perform these types of assessments throughout entire data sets are difficult to create.

Beyond the simple step of examining the probability distribution, we believe it is often best to perform some type of Bayesian analysis (Friston et al., 2003; Koller and Friedman, 2009; Kruschke, 2015). This process involves creating a model and fitting it to the data to obtain model parameters. For instance, if neural connectivity was assessed with transfer entropy, it would probably be best to assume the neurons exist in a network with certain types of excitatory and inhibitory interactions. The existence of a connection between any two neurons could be determined by the existence of a significant transfer entropy result between those neurons. Then, the results of the Bayesian analysis would describe the type of connection that exists between each pair of connected neurons in terms of the assumed model structure. Similar models could be constructed to explain encoding relationships between stimuli and neural activity, relationships between neural activity and behavior, and complex interactions between neural signals such as neural computations.

If the general process we are discussing for analyzing real neuroscience data is to perform an information theory analysis and then build a model, then the demonstration models shown below can be thought of as something similar to this process in reverse. For these demonstrations, the true model that generated the data is known, so we will be able to see how various features of the models correspond to features of the information theoretic results. In a real analysis, one could perform the information theory analysis first, then construct a model that is based on the information theory analysis, and fit the data, for instance.

### Demonstration models

To demonstrate the utility of information theory analyses, we developed several models that primarily use Izhikevich neurons because they are easy to implement, are computationally efficient, and have been widely used in the literature (Izhikevich, 2003, 2007). We wish to emphasize that these models (or simulations) are used here only as a means to produce data very similar to real neural data for demonstration purposes. The purpose of analyzing data from these simulations is not to test novel scientific hypotheses; rather, these simulations are used purely for demonstration purposes. For all models, we attempted to use a realistic number of observations and number of experiments. In all cases, we generated and analyzed 20 models, which is roughly similar to conducting experiments with 20 neural recordings. Also, for all models, we used the equal counts method of binning the data to maximize the entropy of underlying variables and, when neuron spikes are concerned, we examine the information in terms of spike counts (i.e., rate coding). For all models, we include the necessary software to generate and analyze the data used in this article (see *Software*).

#### Small network models of Izhikevich neurons

We used two simple models of individual neurons to produce small networks of neurons (Izhikevich, 2007). We used a model of a regular spiking (RS) neuron that could produce excitatory connections to other neurons, as well as a model of a fast spiking interneuron (FSI) that could produce inhibitory connections to other neurons. These networks allowed us to examine various small circuits and stimulus encoding behaviors. These networks involved one to four neurons, some subset of which were stimulated and/or interconnected. For excitatory neurons in these networks, we used a model governed by the following equations [Eqns. 8.5 and 8.6 in Izhikevich (2007)]:

(27) $$C\frac{\partial v}{\partial t}=k\left(v-v_{r}\right)\left(v-v_{t}\right)-u+I$$

(28) $$\frac{\partial u}{\partial t}=a\left[b\left(v-v_{r}\right)-u\right]$$

(29) $$if\;v\geq v_{peak},\;then\;v\leftarrow c,\;u\leftarrow u+d$$

In Eqns. 27, 28, 29, v represents the membrane potential, u represents the recovery current, C represents the membrane capacitance, $v_{r}$ represents the resting potential, $v_{t}$ represents the instantaneous threshold potential, $v_{peak}$ represents the maximum spiking voltage, k and b are parameters related to the neuron’s rheobase and input resistance, a represents the recovery time constant, c represents the post-action potential voltage reset value, and d represents the net current flow activated during an action potential. One particular combination of parameters has been shown to produce spiking behavior similar to regular spiking neurons (Izhikevich, 2007) (Table 5). In our simulations, we used parameters identical to those discussed in Izhikevich (2007). A similar model has been shown to produce behavior similar to fast spiking interneurons (Izhikevich, 2007) (Eqns. 30, 31, 32, 33):

(30) $$C\frac{\partial v}{\partial t}=k\left(v-v_{r}\right)\left(v-v_{t}\right)-u+I$$

(31) $$\frac{\partial u}{\partial t}=a\left[U\left(v\right)-u\right]$$

(32) $$if\;v\geq v_{peak},\;then\;v\leftarrow c$$

(33) $$\begin{array}{cc} U\left(v\right)=0 & v<v_{b}\\ U\left(v\right)=b{\left(v-v_{b}\right)}^{3} & v\geq v_{b} \end{array}$$

With the exception of k, we used parameters identical to those discussed in Izhikevich (2007) (Table 6). We altered the value of k from 1 to 3.5 to make the neuron more responsive. We felt that this change was appropriate because the purpose of these models was to demonstrate the information theory analyses.

**Table 5.** Regular spiking neuron model parameters.

Parameter	Regular spiking
*C*	100
$v_{r}$	–60
$v_{t}$	–40
$v_{peak}$	35
*k*	0.7
*b*	–2
*a*	0.03
*c*	–50
*d*	100

**Table 6.** Fast spiking interneuron model parameters.

Parameter	Fast spiking interneuron
*C*	20
$v_{r}$	–55
$v_{t}$	–40
$v_{peak}$	25
*k*	3.5
*b*	0.025
*a*	0.2
*c*	–45
$v_{b}$	–55

We simulated inputs from stimuli and presynaptic action potentials using different types of current pulses. Current was injected into neurons from a stimulus using a depolarizing square pulse. Current injected (removed) via an excitatory (inhibitory) connection between neurons was modeled using a positive (negative) gamma function. In all cases, this gamma function had a mean of 30 ms and a standard deviation of 20 ms. The magnitude of the current injected or removed from a connection was constant throughout each simulation in all cases except for the model of sensory habituation. The specific weights for each simulation are listed in Table 7. The models were run using time bins of 0.1 ms. Membrane noise was created using a custom 1/f noise generator to produce spontaneous firing. This noise generator produced Gaussian noise and then filtering the noise in frequency space to produce the appropriate 1/f noise spectrum. It then transformed the noise back to a time-varying signal using an inverse Fourier transform.

**Table 7.** Connectivity weights in small network models.

Figure	Synapse location	Weight (max pA)
11	E1 to E2	200
12	E1 to E2	200
	E1 to I1	200
	I1 to E2	0 to –150
14	E1 to E3	200
15	E1 to I1	50
	E2 to I1	50
	E1 to E3	200
	E2 to E3	200
	I1 to E3	–250
	Background inhibition	–100
16	I1 to E1	–30
	I2 to E1	–30
	Background excitation	0 or 200
17	E1 to E3	100
	E2 to E3	100

#### Large network models of Izhikevich neurons

In addition to small networks of neurons, we used a large 1000-neuron Izhikevich network to examine the behavior of large groups of neurons (Izhikevich, 2003). We used parameters identical to those in the original publication with the exception that we altered the connectivity to be distance dependent and we altered the synaptic weights to produce the same total weights found in the original network. We placed the neurons at random locations on a two-dimensional square (1 spatial unit by 1 spatial unit) with periodic boundary conditions (i.e., a torus). The likelihood $p_{con}\left(r\right)$ that two neurons would be connected decreased exponentially with distance (r) such that at a distance of 0.05 spatial units, the likelihood for two neurons to be connected was 0.5 (Eqn. 34):

(34) $$p_{con}\left(r\right)=0.513e^{-0.513r}$$

In the original network (Izhikevich, 2003), each neuron was equally likely to connect to all others, and the connection weights were uniformly distribution between 0 and 0.5 for connections from excitatory neurons and between –1 and 0 for connections from inhibitory neurons. To compensate for lost connections due to spatial connectivity in our model, we increased the synaptic weights such that the total weights from excitatory and inhibitory neurons considered individually were identical to the original version of the model. The network was stimulated using a square pulse applied to the 40 excitatory neurons closest to a line in the network (i.e., a ring around the torus). In the case of two stimulation simulations, neurons near two parallel lines in the network were stimulated. Each stimulus had a magnitude of 50 and had a duration of 100 ms, with a 1000-ms interstimulus interval.

#### Canonical models

In addition to various networks of Izhikevich neurons, we also employed several models of canonical neuroscience experiments (Bear et al., 2007).

First, using the individual Izhikevich neuron models, we constructed a simulation of sensory habituation in *Aplysia* (Castellucci et al., 1970). This model contained a sensory neuron and a motor neuron, both modeled as RS neurons. The sensory neuron received stimulus pulses and made an excitatory connection on the motor neuron. However, the strength of the current that passed through the model synapse decreased with repeated stimuli. Specifically, the weight of the connection decayed following an exponential function such that the first spike delivered a maximum current of 200 pA, while the last spike delivered a maximum current of 30 pA. This weakening synapse effectively modeled decreased gill and siphon withdrawal reflexes with repeated stimulation in *Aplysia*. Spike counts were binned at 50 ms for information theory analyses of data from these simulations.

Second, we built a simple probabilistic model of center-surround retinal ganglion cells (Kuffler, 1953). These neurons fired preferentially when a light stimulus was applied near the location of the cell but showed decreased firing further from the cell location (ON-center). 300 neurons were placed randomly throughout a two-dimensional square plane (1 unit by 1 unit) with periodic boundary conditions (i.e., a torus). The radius of the ON-region field was 0.1 units, and the outer radius of the OFF-region was 0.3 units. Therefore, cells often had overlapping receptive fields. 400 light dot stimulations were randomly applied throughout the plane. The probability to spike for each neuron was such that it had a background firing rate of 30 Hz, while stimuli in the center region produced firing rates of 100 Hz and stimuli in the surround region produced firing rates of 1 Hz. Spike counts were binned at 25 ms for information theory analyses of data from these simulations.

Third, we constructed a simple probabilistic model of direction-selective motor cortex neurons in primates (Georgopoulos et al., 1982). In this model, a primate moved a cursor on a two-dimensional plane from a center location to one of eight equally spaced locations surrounding the center. The firing of these neurons was modulated such that certain directions of movement were preceded by elevated or depressed firing. The time profile of this change in firing rate was modeled using a Gaussian distribution with a mean of 100 ms before movement onset and a standard deviation of 100 ms. The responsiveness of the neurons was controlled linearly by a parameter r such that highly responsive neurons (r=1) would show a doubled firing rate for the preferred direction, while unresponsive neurons (r=0) would show no change in firing rate based on direction of movement. The probability for a neuron to spike was set such that the neuron had a background firing rate of 50 Hz. Thus, a maximally responsive neuron produce a maximum firing rate of 100 Hz or a minimum firing rate of 0 Hz ∼100 ms before movement onset based on the direction of motion. 20 highly responsive neurons and 100 neurons with randomly selected responsiveness were used, and 150 direction trials were conducted. Spike counts were binned at 25 ms for information theory analyses of data from these simulations.

Finally, we constructed a probabilistic model of place cells in the hippocampus (O’Keefe and Dostrovsky, 1971). This model used a random walk to approximate an animal exploring a square cage. Neurons in the model preferentially fired when the animal was located in certain regions of the cage (place field). The place field of each neuron was randomly selected, and 200 neurons were used in each model. The probability for a neuron to spike was modulated in space using a two-dimensional Gaussian function centered on the place field for that neuron and with a standard deviation of 0.15 spatial units. The probability to spike was set such that when the animal was located at the center of a neuron’s place field, the firing rate was set to 100 Hz and each neuron had a background firing rate of 20 Hz. The animal was allowed to explore the cage for 200 s. Spike counts were binned at 100 ms for information theory analyses of data from these simulations.

### Software

To facilitate the use of information theory analyses in neuroscience, we have created a MATLAB software package (the Neuroscience Information Theory Toolbox) to carry out the analyses discussed in this tutorial article Extended Data. The software uses standard MATLAB functionality throughout, so once the user’s data are rendered as MATLAB variables, the remainder of the analysis can be conducted entirely in MATLAB. Our overall goal was to create software that functioned like any other basic MATLAB functions. In short, if you know how to manipulate matrices and use built-in functions in MATLAB, you will be able to quickly use this software. The analysis software is thoroughly documented within each function similar to built-in MATLAB functions, and overall guidance is supplied via a README file. Numerous simple demonstrations are included in addition to the software to generate the data and perform information theory analyses for all of the simulations discussed in this article. These simple demonstrations serve to highlight the coding necessary to implement various parameters and settings associated with different analyses. Furthermore, the software is capable of performing significance testing using both the real data and predefined null models (see *Significance Testing*), though this functionality was not employed in the simulations presented herein. Finally, basic functions are provided so the user can build more complicated analysis software to suit their needs, as well as macro-style functions that can be quickly and easily used to perform information theory analyses.

In fact, the generality of the software means that the software can be used to analyze data from disciplines other than neuroscience. We chose to use the word “neuroscience” in the name of the software package to highlight its intended role, but it can just as easily be applied to data from economics, physics, computer science, or sociology as it can to data from neuroscience.

While we feel our software package fills a valuable role in the field, we wish to emphasize that other excellent information theory software packages exist and that these packages may be more useful depending on the desired application (Ince et al., 2010; Quiroga and Panzeri, 2013; Lizier, 2014; Szabo, 2014; Moore et al., 2017). We explored 10 other available software packages and documented their important features to aid readers in comparing software options (Table 8). We found that many of these other packages are focused on a narrower type of analysis. Several software packages have been introduced to calculate transfer entropy (Ito et al., 2011; Lindner et al., 2011; Montalto et al., 2014; Pastore et al., 2016), often with the emphasis on estimating neural connectivity. Also, several software packages have focused on estimating entropy and mutual information using more advanced techniques (e.g., binless and kernel estimation techniques, as well as bias correction) than those presented herein to address problems surrounding continuous data and binning (Goldberg et al., 2009; Ince et al., 2009; Magri et al., 2009; Lindner et al., 2011; Lizier, 2014). In total, these software packages are capable of providing superior analyses of certain types of data, though their underlying assumptions (e.g., Gaussian distributed data in some cases) must be carefully weighed. Furthermore, it should be noted that, to the best of our knowledge, no such advanced estimation techniques have been extended to the partial information decomposition. We intentionally chose to not include bias correction algorithms in our software because of the presence of significance testing algorithms. We feel this aligns better with the introductory goals of this paper and typical neuroscience experiments where two values are compared while the absolute value of the result is frequently less important.

**Table 8.** Information theory analysis software package comparisons.

Software package	Information measures	Data types	Dynamic information capabilities? (ensemble methods from multiple trials)	Significance testing?	Advanced probability distribution estimation methods and/or bias correction	Language
Neuroscience Information Theory Toolbox	Entropy, mutual information, transfer entropy, partial information decomposition, information transmission, conditional variants	Discrete and continuous	Yes	Yes	No	MATLAB
JIDT (Lizier, 2014)	Entropy, mutual information, transfer entropy, information storage, conditional variants	Discrete and continuous	Yes	Yes	Yes	JAVA (with Python and MATLAB functionality)
Inform (Moore et al., 2017)	Entropy, Mutual Information, Transfer Entropy	Discrete	Yes	Not directly	No	C (with Python functionality)
Transfer Entropy Toolbox (Ito et al., 2011)	Transfer entropy	Spike trains only	No	Not directly	No	MATLAB
Trentool (Lindner et al., 2011)	Transfer entropy	Primarily continuous	Yes	Yes	Yes	MATLAB
MuTE (Montalto et al., 2014)	Transfer entropy	Primarily continuous	No	Yes	Yes	MATLAB
ToolConnect (Pastore et al., 2016)	Entropy, transfer entropy	Spike trains only	No	Yes	No	C++
STAToolkit (Goldberg et al., 2009)	Entropy, mutual information	Spike trains only	Not directly	Yes	Yes	MATLAB
PyEntropy (Ince et al., 2009)	Entropy, mutual information	Discrete and continuous	Not directly	Not directly	Yes	Python
Information Breakdown Toolbox (Magri et al., 2009)	Entropy, mutual information, breakdown information	Discrete and continuous	Not directly	Not directly	Yes	MATLAB
ITE Toolbox (Szabo, 2014)	Entropy, mutual information	Discrete and Continuous	Not directly	Not directly	Yes	MATLAB and Python
dit (dit-contributors, 2018)	Entropy, mutual information, and many more	Discrete	Not directly	Not directly	No	Python

We examined ten other information theory software packages and recorded important features for users. Many packages are either focused on transfer entropy alone or entropy and mutual information calculations. Many packages include advanced estimation and bias correction techniques, unlike the neuroscience information theory toolbox.

We attempted to perform computation speed comparisons with all of the software packages listed in Table 8. Unfortunately, we were only able to perform comparisons of identical analyses using the JIDT Toolbox (Lizier, 2014; 10 times faster than the Neuroscience Information Theory Toolbox) and the Transfer Entropy Toolbox (Ito et al., 2011; 100 times faster than the Information Theory Toolbox). This speed performance was not surprising given the focus of the Neuroscience Information Theory Toolbox on flexibility and standard MATLAB functionality. We were unable to test the other packages for two main reasons. First, several packages require the use of more advanced probability estimation techniques that are not available in the Neuroscience Information Theory Toolbox (Lindner et al., 2011; Montalto et al., 2014; Szabo, 2014). As a result, these packages took significantly longer to perform a given information theory calculation, but this was not a fair comparison due to the additional calculations performed by the other software package. Second, several software packages could not be made to run on our machines or required programming languages with which we are not familiar (Goldberg et al., 2009; Ince et al., 2009; Magri et al., 2009; Pastore et al., 2016; Moore et al., 2017; dit-contributors, 2018). Obviously, our inability to test the software may be a larger reflection on our programming abilities (or our lack of available time to devote to learning new languages) than on the quality of the software itself. However, we feel these are still relevant details for readers who may find themselves in similar positions. We anticipate that many of these software packages are orders of magnitude faster than the Neuroscience Information Theory Toolbox based on the languages in which they are written or their use of mex files in MATLAB.

In summation, we highly recommend that the interested reader pursue these other software packages if his or her research question better aligns with the goals of another software package. While many different software packages exist, we found that most of them differ in subtle but important ways regarding functionality, data types, purpose, and programming language. For instance, the dit toolbox lacks methods for handling experimental data, but it maintains a very large set of available information measures for discrete probability distributions (Dit-contributors, 2018). For the purposes of analyzing experimental data in a neuroscience context, we found the JIDT software package to be very helpful (Lizier, 2014). It possesses almost all of the information measured discussed here, data can be analyzed via an intuitive GUI, it can implement more complicated information estimation techniques, and, though it is written in JAVA, implementation in MATLAB is straightforward in most respects. JIDT does have some issues with importing/organizing data from MATLAB, different information measures, and interpretation of time-dependent information measures, but we would probably recommend it for most users as the next software to use after the Neuroscience Information Theory Toolbox. At the very least, we hope that this paper and our software package provide the reader with a useful introduction to information theory and information theoretic analyses of neuroscience data.

#### Software accessibility

The MATLAB software used in this tutorial is part of the Extended Data.

## Results

### Single neuron stimulus encoding

**Figure 10.**
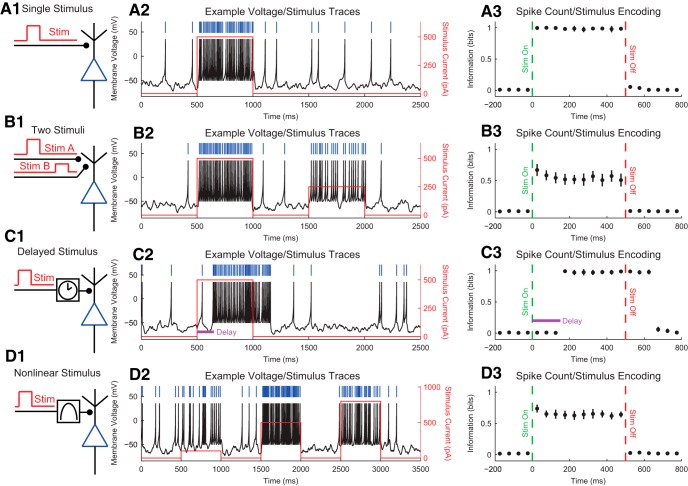
Single neuron stimulus encoding is captured in a variety of situations. ***A***, Stimulus on versus stimulus off. ***B***, Strong stimulus versus weak stimulus. ***C***, Stimulus delay. ***D***, Nonlinearly filtered stimulus. ***1***, Explanatory diagrams. ***2***, Neuron firing rates were modified by the application of a depolarizing square pulse. Blue lines: spikes; ***A2*** and ***B2*** involved the application of a strong stimulus and a zero or weak stimulus, respectively. ***C2*** involved a delay between the application of the stimulus and it being received by the neuron. ***D2*** involved a nonlinear filter of the stimulus that weakened the strongest applied stimulus and strengthened the weakest applied stimulus. ***3***, Stimulus encoding through time as measured by mutual information between the spike count of the neuron and the stimulus state [(***A3*** and ***C3***): on/off (***B3***): strong/weak (***D3***): weak/medium/strong, dots: mean, error bars: standard deviation across models (n=20)]. In all cases, large amounts of mutual information were observed between the spike count and the stimulus state during the stimulus, but not otherwise (accounting for the delay in ***C***).

To demonstrate a possible use for mutual information (Eqn. 16), we will first examine stimulus encoding by an individual neuron (Fig. 10). In these examples, we describe various scenarios where information theory can be used to identify neurons that encode a stimulus (or some other variable). Note that similar techniques could be used to identify other signals that encode a stimulus. In the simplest case (Fig. 10*A*), a square wave current pulse was applied to the neuron (Fig. 10*A2*). The spike count during the pulse (e.g., 500–1000 ms, Fig. 10*A2*) was compared using mutual information to the spike count during a period with no pulse (e.g., 1500–2000 ms, Fig. 10*A2*). One variable was the stimulus state (on versus off) and the other variable was the spike count in 50-ms bins, which was then binned into two equal count bins. As expected, very little mutual information was observed before the start of the stimulus (Fig. 10*A3*, 0 ms). No information was observed during this time period because there was no difference in firing rate between the stimulus on and off time periods (for instance, compare ∼400 ms and ∼1400 ms in Fig. 10*A2*). Then, during the stimulus, the spike count of the neuron provided a great deal of information about the stimulus state (compare ∼600 ms and ∼1600 ms in Fig. 10*A2*). Finally, when the stimulus ended, the mutual information dropped to near zero (compare ∼1100 ms and ∼2100 ms in Fig. 10*A2*).

A similar pattern was observed when two stimuli were applied to a neuron (Fig. 10*B1*). In this example, the two stimulus states were strong and weak (Fig. 10*B2*), but the spike count variable maintained a similar structure. Here, the mutual information increased during the stimulus (Fig. 10*B3*), but the increase was not as strong as in the previous example because the spike counts of the neuron were not able to differentiate the two stimuli states as accurately.

When a delay in the stimulus was used (Fig. 10*C*), patterns of spiking and mutual information that were similar to Fig. 10*A* were observed, except that the spiking and mutual information were delayed. Finally, even when a nonlinear filter was applied to three stimuli (Fig. 10*D*), mutual information was still able to detect encoding of the stimulus by the neuron (Fig. 10*D3*).

Please note that we intentionally used a strong stimulus in this example (and many subsequent examples) to make the interactions readily apparent. They are so strong in fact that in the examples with two stimuli, it would likely be possible to observe a significant difference in spike rate between the stimuli with a simple *t* test (see Fig. 5). However, real data are not likely to produce such strong effects, information theory allows for the quantification of the effect sizes, and information theory easily allows for the analysis of cases with more than two stimuli.

### Two-neuron information transmission

**Figure 11.**
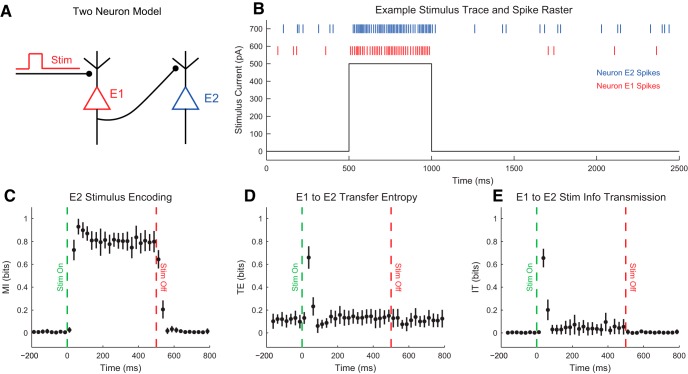
Information transmission between neuron peaks at the onset of transmission. ***A***, An excitatory neuron (E1) received a stimulus and then sent current to a second excitatory neuron (E2). ***B***, Both E1 and E2 spiked during the stimulus, though E1 started spiking earlier. ***C***, Mutual information between E2 and the stimulus state (on/off). E2 encoded the spiking state throughout the stimulus. ***D***, Transfer entropy from E1 to E2 peaked immediately following the onset of the stimulus and was nonzero before, during, and after the stimulus. This elevated transfer entropy was due to the constant existence of the connection. ***E***, Information transmission from E1 to E2 about the stimulus state (on/off) peaked at the onset of the stimulus, was nonzero throughout the stimulus, but was near zero otherwise. [For all information plots, dots: mean, error bars: standard deviation across models (n=20)].

The simple one-neuron simulations shown in Fig. 10 above can easily be expanded to two neurons to demonstrate information transmission (Fig. 11). These examples demonstrate how information theory can be used to identify connected neurons and the variables about which they communicate. Note that similar analyses could be used to identify communication between other signals. In this simulation, a single excitatory neuron (E1) is stimulated by a square wave depolarizing pulse and makes a synapse on a second excitatory neuron (E2; Fig. 11*A*). As can be seen in an example spike raster, both E1 and E2 spiked more frequently during the stimulation, and a slight delay was observed for E2 relative to E1 (Fig. 11*B*). As expected from the example spike raster, E2 still encodes the stimulus by firing more frequently when the stimulus was on in comparison to when it was off (Fig. 11*C*, mutual information between spike count in 25-ms bins and stimulus state (on versus off)).

Next, we examined the amount of information carried by the synapse using transfer entropy (Fig. 11*D*). In this case, transfer entropy measures the amount of information that the spike count of E1 provides about the spike count of E2 in the next time bin beyond the information provided by the spike count of E2 in the past (Eqn. 19). It does not directly take account of the stimulus. Because the neurons are always connected, and individual background spikes in E1 can influence E2, a steady nonzero transfer entropy was observed for time periods when the stimulation was never applied (i.e., before the stimulus turned on and after the stimulus turned off). However, immediately following the onset of the stimulus, a large peak in the transfer entropy was observed because the spiking state of E1 just as the stimulus began largely affected the state of E2 in a way that could not be predicted based on the past state of E2 alone. While the stimulus was on, the past state of E2 provided a great deal of information about its future state because the stimulus was constant. Therefore, the transfer entropy value returned to its nonzero background level for the remainder of the stimulus. A similar peak near the end of the stimulus was not observed because E2 returned to its background low firing rate, which was not distinguishable between stimulus on and stimulus off trials. Therefore, the future state of E2 had low entropy, so transfer entropy was also low.

Finally, we used the information transmission (Eqn. 25) to measure the amount of information about the stimulus transmitted from the spike count of E1 to the spike count of E2 (Fig. 11*E*). Information transmission was near zero during the periods when the stimulus was never on, unlike transfer entropy. Similar to transfer entropy, a large peak was observed in the information transmission immediately after the stimulus began. This indicates that the synapse was carrying a large amount of information about whether the stimulus was on or off as the firing rate of E2 was increasing dramatically. However, once the firing rate increased, the information transmission decreased to a low nonzero value. As with transfer entropy, the information transmission was low during this period because the past state of E2 provided a great deal of information about the state of the stimulus. However, unlike transfer entropy, the information transmission was zero during time periods when the stimulus was never on because no information about the stimulus was transmitted during this time.

### Inhibition modulated encoding

**Figure 12.**
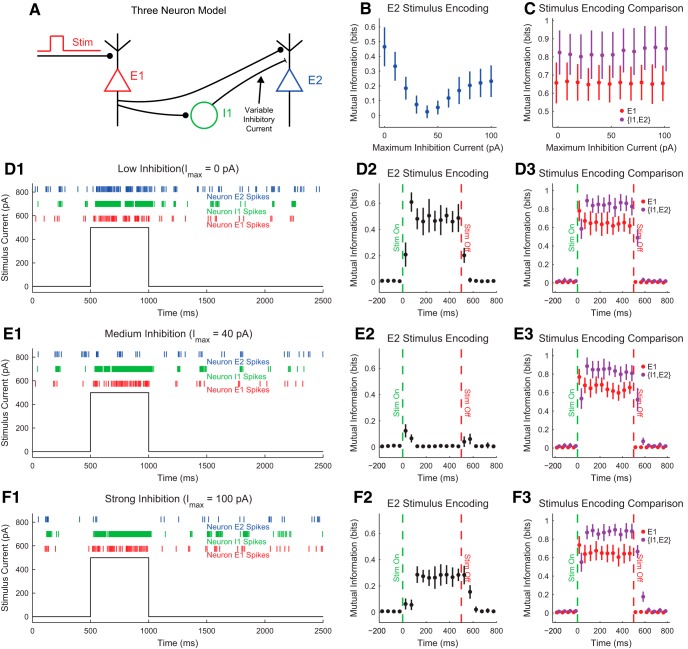
Inhibition can modulate stimulus encoding modalities. ***A***, Excitatory neuron E1 received stimulus current and sent current to inhibitory neuron I1 and excitatory neuron E2. Neuron I1 also inhibited neuron E2. ***B***, Average mutual information during stimulus between the spike count of E2 and the stimulus state (on/off) as a function of inhibition current from I1 to E2. Note the local maxima in encoding for low inhibition and high inhibition. Also, note that mutual information is able to detect both firing rate increases and decreases, though firing rate decreases provide less information. ***C***, Average mutual information during stimulus between the stimulus state (on/off) as a function of inhibition current from I1 to E2 for E1 alone and for I1 and E2 jointly. Note that I1 and E2 jointing encoded the stimulus state for all inhibition levels better than E1 alone, despite the fact that only E1 received the stimulus current. ***D***, Weak inhibition. ***E***, Medium inhibition. ***F***, Strong inhibition. **(1)** Example spike rasters. **(2)** Mutual information between the stimulus state (on/off) and neuron E2. **(3)** Mutual information between the stimulus state (on/off) and E1 alone or I1 and E2 jointly. In ***D***, neuron E2 encoded the stimulus state by increasing firing during the stimulus on state. In ***E***, the inhibition and excitation balanced to render neuron E2’s firing rate unchanged by the stimulus. In ***F***, neuron E2 encoded the stimulus state by decreasing firing during the stimulus on state. [For all information plots, dots: mean, error bars: standard deviation across models (n=20)].

The impact of inhibition on encoding can be demonstrated by adding an inhibitory neuron to the circuit described in Fig. 11 and examining similar spike count encoding. This example demonstrates how information theory can be used to study the influence of excitation and inhibition on information encoding. By adding an inhibitory neuron (I1) after E1 and varying the strength of its synapse on E2 (Fig. 12*A*), various encoding strategies were observed. First, when increasing the strength of the inhibitory synapse, a minimum in stimulus encoding by E2 was observed (Fig. 12*B*, mutual information between E2 spike count in 50-ms bins and stimulus state (on versus off). This behavior can be better understood by examining example spike rasters from a weak inhibition model (Fig. 12*D1*), a medium inhibition model (Fig. 12*E1*), and a strong inhibition model (Fig. 12*F1*). The weak inhibition model produced elevated firing in E2 during the stimulus, thus producing encoding (ON encoding; Fig. 12*D2*). The strong inhibition model produced depressed firing in E2 during the stimulus, also producing encoding (OFF encoding; Fig. 12*F2*). When the inhibition was correctly balanced, the firing rate of E2 was generally unchanged by the stimulus, producing little encoding (Fig. 12*E2*). However, in all inhibition cases, higher encoding was observed by I1 and E2 jointly in comparison to E1 (Figs. 12*C*, *D3*, *E3*, and *F3*). In this case, the joint encoding was measured by calculating the mutual information between the stimulus state and the joint state of the spike counts of the I1 and E2 neurons (Eqn. 17). This result is especially noteworthy because I1 and E2 only receive information about the stimulus via E1. This result highlights the importance of the encoding power gained by increasing the number of encoders, especially in a noisy system of neurons.

### Information transmission and encoding in a large network

**Figure 13.**
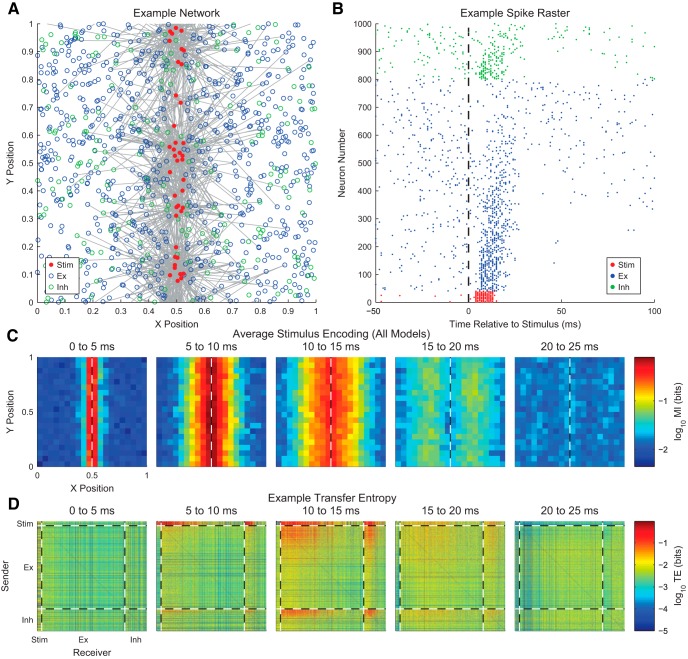
Activity waves carry stimulus information and transmit information. ***A***, Example 1000 neuron Izhikevich network on a 2-D surface with periodic boundary conditions and distance dependent connectivity. 40 neurons near the center line were stimulated. Only connections from stimulated neurons are shown to improve clarity (gray lines). ***B***, Example spike raster sorted by distance from the *x* = 0.5 line. Following the application of the stimulus, a wave of activity propagated outwards from the center. ***C***, Average mutual information across all models (n=20) between the stimulus state (on/off) and the neurons as a function of neuron position. Note that the encoding spreads outwards from the center line of the network. ***D***, Example transfer entropy between neurons as a function of time from stimulus. The nonstimulus neurons are sorted by distance from the line *x* = 0.5. Note that transfer entropy first appears from stimulated neurons to nearby nonstimulated neurons (5–10 ms), then appears from nearby nonstimulated neurons to more distant neurons (10–15 ms).

To move beyond simple circuits of a few neurons, we examined a large 1000-neuron network model (Izhikevich, 2003; Fig. 13). This example demonstrates how information theory can be used to identify information encoding and transmission in larger networks. Excitatory (800) and inhibitory (200) neurons were randomly arranged on a 2-dimensional plane with periodic boundary conditions and preferentially connected to other nearby neurons (Fig. 13*A*). A small number of excitatory neurons (40) near the center line of the plane were stimulated with a depolarizing square pulse similar to previous simulations. Immediately after the pulse, a wave of activity propagated outward from the center line of the plane (Fig. 13*B*). As expected this wave of activity carried information about whether the stimulus had been applied or not [Fig. 13*C*, mutual information between stimulus state (on versus off) and spike count in 5-ms bins]. Furthermore, elevated transfer entropy was observed within the wave and directed outward as the wave moved outward from the center line (Fig. 13*D*, neuron spike counts in 5-ms bins).

The result that transfer entropy is changing as the wave of activity spreads through the network demonstrates the difference between physical (or structural) connectivity and functional or effective connectivity (Sporns, 2007, 2013). In this example, the underlying physical connectivity of the network did not change at any point in the simulation, but the transfer entropy (which measures functional or effective connectivity) did change. As an analogy, physical connectivity tells you where the pipes are in the house, while functional connectivity tells you where the water flows. When there is little activity in the network, it is possible for physically connected neurons to not produce significant amounts of transfer entropy if the driving neuron is not active. However, once the driving neuron becomes active, it is possible to detect significant transfer entropy. The crucial difference between these two types of connectivity is of vital importance and has been widely discussed in the literature (Sporns, 2007, 2013; Bullmore and Sporns, 2009; Friston, 2011; Schölvinck et al., 2013). Therefore, this distinction should be considered in an analysis of this type. Obviously, both physical and functional connectivity are interesting and important. However, this example demonstrates that transfer entropy is a measure of functional or effective connectivity, not physical connectivity.

### Small circuit example: unique information

**Figure 14.**
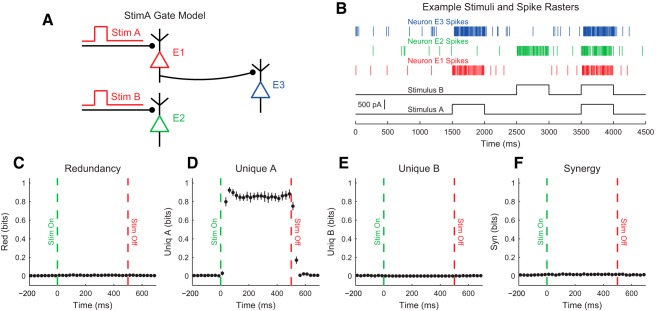
Unique information represents encoding about one stimulus in a joint set. ***A***, Excitatory neuron E1 received input current from stimulus A, while excitatory neuron E2 received input current from stimulus B. Only E1 sent current to excitatory neuron E3. ***B***, Example spike raster with stimuli. As expected, stimulus A caused neuron E1 to fire, which caused neuron E3 to fire. ***C–F***, PID values between the spike count of E3 and the stimuli states (on/off). Neuron E3 encoded only the state of stimulus A, so E3 uniquely encoded stimulus A. [For all information plots, dots: mean, error bars: standard deviation across models (n=20)].

To examine converging information flows and computation, we created a small network similar to that described in Fig. 11, except that a second excitatory neuron driven by a different stimulus was added (Fig. 14*A*). This example demonstrates how unique information can be used to identify information transmission in a system with multiple neurons and possible connections. In this case, because the second stimulated neuron (E2) did not make a synapse on E3, stimulus B did not influence the activity of E3 (Fig. 14*B*). When the partial information decomposition was applied to these data with the states of the two stimuli as the X variables and the spike count of E3 in 25-ms bins as the Y, only unique information about stimulus A was present in E3 (Fig. 14*C*–*F*). This is expected because no information about stimulus B was present in the spiking activity of E3.

### Small circuit example: synergy

**Figure 15.**
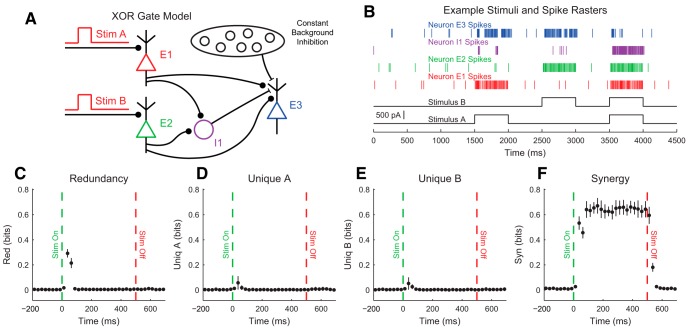
Synergy represents encoding simultaneous information about both stimuli. ***A***, Neuron E3 received excitatory inputs from neurons E1 and E2, both of which received stimulation. Neurons E1 and E2 also sent current to inhibitory neuron I1, which inhibited E3. Neuron E3 also received constant background inhibition from other neurons. ***B***, Example spike rasters. Neurons E1 and E2 fired when their respective stimulus is applied. Note that neuron E3 only fired when either E1 or E2 was active, but not both due to inhibition from I1. ***C–F***, PID values between the spike count of E3 and the stimuli states (on/off). Neuron E3 showed sustained synergy because it encoded information about the simultaneous states of stimuli A and B. [For all information plots, dots: mean, error bars: standard deviation across models (n=20)].

To create a similar small model circuit that produced high synergy, we added an inhibitory neuron (I1) and constant weak background inhibition (Fig. 15*A*). This example demonstrates how information theory can be used to identify and quantify a complex interaction among several neurons. The behavior of this system was similar to an XOR logic gate in that E3 showed elevated spiking when only one of the stimuli was on, but not both (Fig. 15*B*). We then applied the partial information decomposition analysis with the stimuli as the X variables and the spike count of E3 in 25-ms bins as the Y variable (Fig. 15*C*–*F*). As expected, we found large amounts of synergy because the spiking activity of E3 provides information about the activity of both stimuli together, but it does not provide information about either stimulus alone. Put another way, the state of each stimulus in isolation does not determine the spiking activity of E3. Rather, only simultaneous knowledge of both stimuli will determine the state of E3.

### Small circuit examples: synergy and redundancy

**Figure 16.**
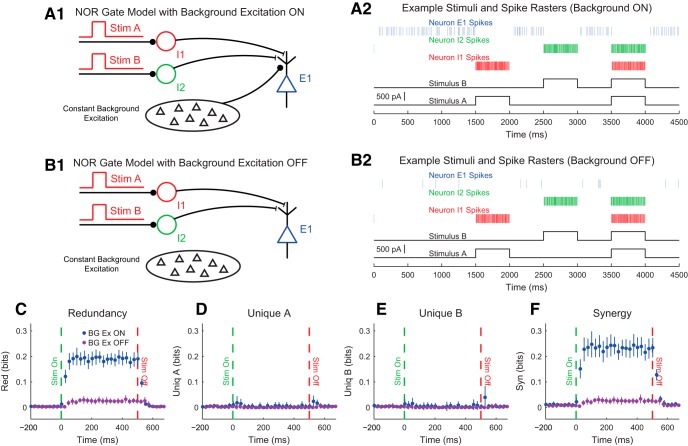
Varying background activity can produce NOR-Gate like activity and modulate redundancy and synergy. ***A1***,***B1***, Inhibitory neurons I1 and I2 received unique stimuli and inhibited neuron E1. In ***A1***, neuron E1 also received background constant excitation, but not in ***B1***. ***A2***,***B2***, Example spike rasters. In ***A2***, the background excitation made E1 perform a NOR operation (E1 fired when neither ***A*** nor ***B*** is on). ***C–F***, PID values between the spike count of E1 and the stimuli states (on/off). Neuron E1 showed sustained synergy and redundancy with the background excitation on, but little encoding with background excitation off. Synergy and redundancy were observed because the encoding provided simultaneous information about both stimuli for some cases, but not all cases. [For all information plots, dots: mean, error bars: standard deviation across models (n=20)].

The interplay between inhibition and excitation can influence the presence of both synergy and redundancy, as shown by small circuit models with two stimulated inhibitory neurons, background excitation, and a postsynaptic excitatory neuron (Fig. 16*A1* and *B1*). This example demonstrates how information theory can be used to differentiate between different connectivity structures among neurons across different scales. When background excitation was present to increase the background firing rate of the excitatory neuron, the influence of the stimuli was shown via the reduction in spiking behavior (Fig. 16*A2*). The behavior of this circuit is similar to a NOR logic gate because the spiking activity of E1 is elevated only when neither stimulus A nor B is on. Similar reductions in firing rate were not observed when the background excitation was not present (Fig. 16*B2*). We then applied the partial information decomposition analysis with the stimuli as the X variables and the spike count of E1 in 25-ms bins as the Y variable (Fig. 16*C*–*F*). When the background excitation was on, both synergy and redundancy were observed, but when the background excitation was off, all information values were near zero. This second result makes sense because E1 provided little information about the stimuli either alone or jointly when the background excitation was off. When the background excitation was on, E1 provided some information about both stimuli individually, but also some information about their joint state, resulting in synergy and redundancy. Said another way, it requires simultaneous information about stimuli to know if E1 had high spiking activity, but if either stimuli was on, it would be known that the spiking activity of E1 would be low.

**Figure 17.**
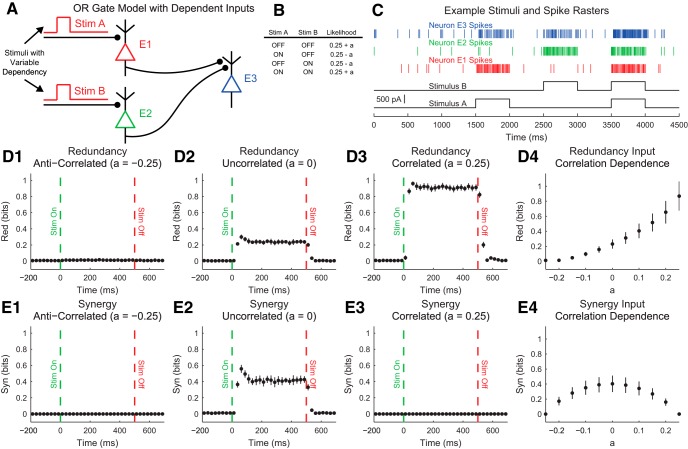
Input correlation affects synergy and redundancy. ***A***, Excitatory neurons E1 and E2 received stimuli and sent current to neuron E3. ***B***, The correlation between the stimuli can be modulated by the parameter a (a=−0.25 implies anticorrelation, a=0 implies uncorrelated, and a=0.25 implies correlation). ***C***, Example spike raster in the uncorrelated case (all four stimuli combinations are equally likely). Note that the correlation affected the number of times each stimuli pattern is observed, but not the spiking activity that resulted from a given stimulation pattern. PID redundancy (***D***) and synergy (***E***) between neuron E3 spike count and the stimuli state. ***1***, Anticorrelated stimuli. ***2***, Uncorrelated stimuli. ***3***, Correlated stimuli. ***4***, Average information value during stimulation as a function of correlation parameter a. In the anticorrelated case, neuron E3 did not encode the stimuli. In the uncorrelated case, both synergy and redundancy were present. In the correlated case, only redundancy was present. [For all information plots, dots: mean, error bars: standard deviation across models (n=20)].

In the previous two stimuli examples, the stimuli were independent. However, if the stimuli are dependent, additional changes in synergy and redundancy can be observed (Fig. 17). This example demonstrates how information theory can be used to analyze the effects of varying stimuli on a system of neurons. This simulation contained three excitatory neurons, two of which received stimuli and then made synapses on the third excitatory neuron (E3; Fig. 17*A*). The relationship between the stimuli was modulated by a parameter a (Fig. 17*B*) such that a<0 implied anticorrelation between the stimuli and a>0 implied correlation between the stimuli. In the uncorrelated case, E3 produced elevated spiking activity when either stimuli was on, similar to an OR logic gate (Fig. 17*C*). We applied the partial information decomposition analysis with the stimuli as the X variables and the spike count of E3 in 25-ms bins as the Y variable using a variety of a values (Fig. 17*D*,*E*). When the stimuli were anticorrelated, E3 always showed elevated spiking activity, so it provided no information about the stimuli (Fig. 17*D1* and *E1*). When the stimuli were uncorrelated, redundant and synergistic information was observed in the system (Fig. 17*D2* and *E2*) similar to the behavior seen in Fig. 16. When the stimuli were correlated, only redundant information was observed in the system (Fig. 17*D3* and *E3*). In this case, the stimuli provided the same information about the spiking state of E3 because the stimuli behaved identically (see Pica et al. (2017) for a recent further possible refinements of redundancy). As the correlation tuning factor was changed, the synergy was found to peak near an uncorrelated system, while redundancy peaked when the stimuli were most correlated (Fig. 17*D4* and *E4*).

### Synergy, redundancy, and unique information in a large network

**Figure 18.**
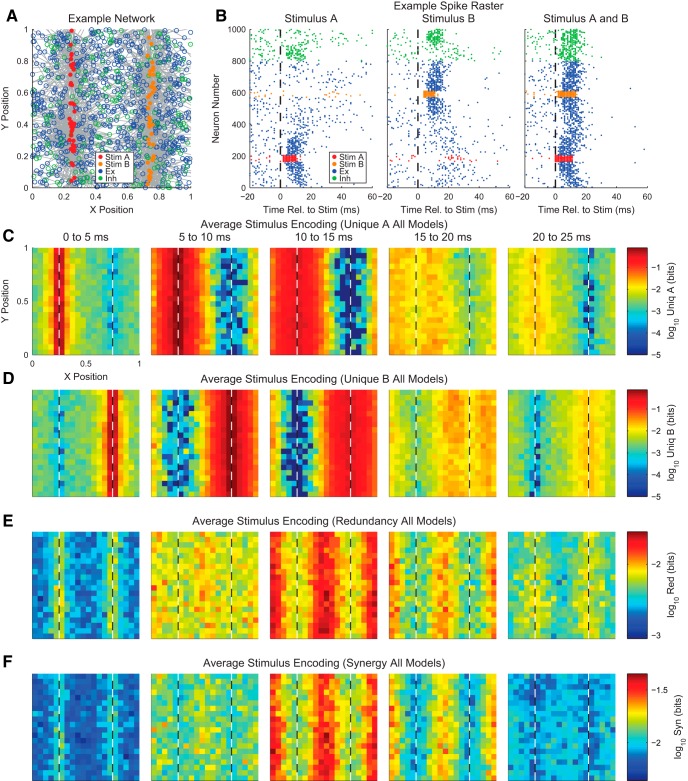
PID reveals redundant and synergistic encoding at activity wave collision points. ***A***, Example 1000 neuron Izhikevich network on a 2-D surface with periodic boundary conditions and distance dependent connectivity. 40 neurons near the line *x* = 0.25 (*x* = 0.75) received stimulus A (B). Only connections from stimulated neurons are shown to improve clarity (gray lines). ***B***, Example spike rasters sorted by *x* position. Following the application of stimulus, a wave of activity propagated outwards from the stimulation points. (No stimulus spike rasters not shown.) ***C***,***D***, Average PID values across all models (n=20) between the spike count of each neuron and the stimuli states (on/off) as a function of location. Neurons closest to the stimulation lines showed large amounts of unique encoding for the corresponding stimulus (***C*** and ***D***). Neurons between the stimulus locations (where the activity waves collided) showed high levels of synergy and redundancy (***E*** and ***F***).

We modified the large 1000-neuron network shown in Fig. 13 to be stimulated at two points to demonstrate synergy, redundancy, and unique information in a large network (Fig. 18*A*). This example demonstrates how information theory can be used to analyze complex encoding of multiple stimuli in a large network of neurons. As expected, stimulation of the network produced activity waves that traveled outward from the stimulation sites (Fig. 18*B*). We applied the partial information decomposition analysis with the stimuli as the X variables and the spike count of a neuron in 5-ms bins as the Y variable (Fig. 18*C*–*F*). We examined the information values as a function of time after the stimulus and the location of the neuron in the network. We observed synergy and redundancy where the activity waves collided (near x=0 and x=0.5; Fig. 18*E*,*F*). We observed unique information for each stimulus near the location in the network where that stimulus was applied (either x=0.25 or x=0.75; Fig. 18*C*,*D*).

### Aplysia stimulus response habituation

In the previous demonstrations, we considered various neural circuits and models designed specifically to demonstrate applications of various information theory measures. We now turn to four simulations designed to connect with canonical experiments in neuroscience to demonstrate the role information theory can play in these different contexts.

First, we examined a simple model of sensory habituation in *Aplysia* (Castellucci et al., 1970; Bear et al., 2007). The gill and siphon of the sea snail *Aplysia* are highly sensitive and will withdraw when touched. However, if the gill and siphon are repeatedly stimulated, the withdrawal reflex decreases in strength (i.e., the sensory response is habituated). The neural circuitry underlying this reflex is relatively simple. A sensory neuron responds to stimulation of the gill and siphon. This sensory neuron then makes a synapse on a motor neuron that controls the withdrawal muscle. It has been shown (Castellucci et al., 1970; Bear et al., 2007) that the habituation process is due to changes in the synapse between the sensory neuron and the motor neuron.

**Figure 19.**
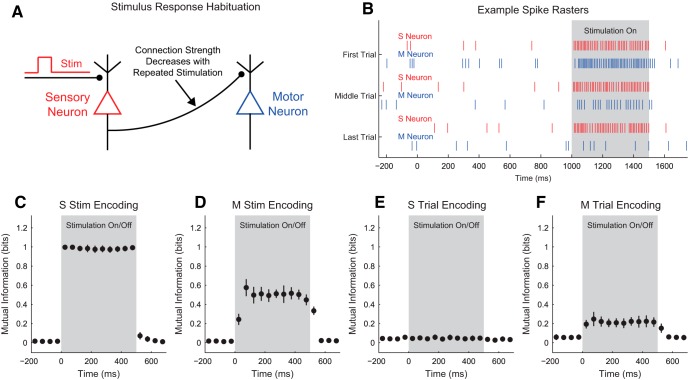
Habituated motor neuron encodes stimulus type and number. ***A***, A sensory neuron (S) was stimulated and sent current to a motor neuron (M). The strength of the synapse weakened with repeated stimulation of S. ***B***, Example spike rasters. In the first trial, stimulation of the sensory neuron caused elevated spiking of the sensory neuron and the motor neuron. However, by the last trial, stimulation of the sensory neuron caused elevated spiking of only the sensory neuron. ***C***,***D***, Mutual information between a neuron’s spike count and the stimulus state. The weakening synapse caused weaker encoding by the motor neuron, though it did still encode the stimulus. ***E***,***F***, Mutual information between a neuron’s spike count and the trial number (e.g., early/late). Because the motor neuron’s activity changed with trial, the motor neuron encoded the trial number. [For all information plots, dots: mean, error bars: standard deviation across models (n=20)].

To model sensory habituation, we used a circuit that was very similar to the circuit shown in Fig. 11, except that the excitatory connection from the stimulated neuron (Sensory (S) Neuron) to the postsynaptic neuron (Motor (M) Neuron) decreased in strength with repeated stimulation via an exponential decay (Fig. 19*A*). This circuit and changing synaptic strength produced decreasing motor neuron response to the stimulus with successive stimulation of the sensory neuron (Fig. 19*B*). As expected, when the mutual information between the stimulus (on versus off) and the spike count of the neurons in 50-ms bins was calculated, the sensory neuron encoded the stimulus very well (Fig. 19*C*), but the motor neuron also encoded the stimulus, though to a smaller degree (Fig. 19*D*). However, when the mutual information between the spike count of each neuron and the trial number (e.g., early trial versus late trial) was assessed, we found no trial number encoding by the sensory neuron (Fig. 19*E*), but the motor neuron did weakly encode the trial number (Fig. 19*F*). This result is because the sensory neuron does not change behavior through the stimulation trials, so it cannot encode trial number. Conversely, the motor neuron does exhibit altered behavior with successive trials, so it does encode the trial number. The use of information theory in this analysis clearly identified the sensory and motor neurons, quantified the different amount of stimulus encoding, and identified changing motor neuron behavior with repeated stimulation.

### Center-surround retinal ganglion cells

The second canonical neuroscience experiment we simulated was the center-surround receptive fields of retinal ganglion cells (Kuffler, 1953; Bear et al., 2007). These retinal ganglion cells exhibit elevated firing when a light stimulation is applied near the center of the receptive field, but exhibit decreased firing when the light stimulation is applied slightly farther away from the center of the receptive field (i.e., a so-called ON-center cell). When a light stimulus is applied far from the center of the receptive field, no change in the firing of the cell is observed. Thus, the receptive field of these cells is a circular region with elevated firing surrounded by an annular region with depressed firing. This type of cell is capable of providing information about the location of a light stimulus. Furthermore, two cells of this type are capable of encoding the light stimulus location in complex ways that are dependent on the relative positions of their receptive fields.

**Figure 20.**
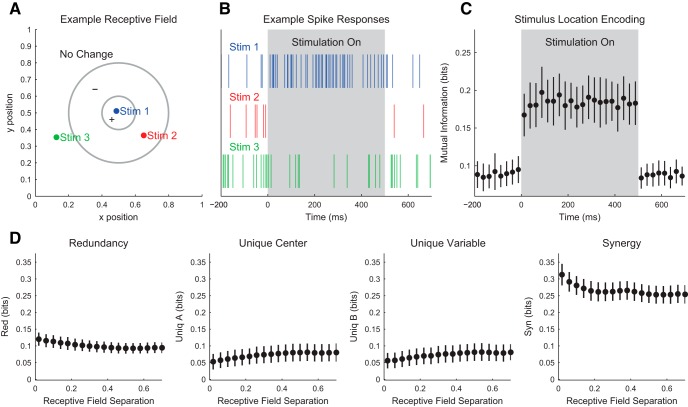
Model center-surround retinal ganglion cells jointly encode stimulus location synergistically and redundantly. ***A***, Example receptive field for a neuron in a 2-D plane with periodic boundary conditions showing stimulation locations that increase (+), decrease (–), or do not change the firing of the neuron. ***B***, Example spike rasters for the stimuli and receptive field shown in ***A***. Stim 1 occurred in the center of the receptive field and increased firing. Stim 2 occurred in the periphery of the receptive field and decreased firing. Stim 3 occurred outside the receptive field and did not affect firing. ***C***, Mutual information between the stimulus location and the spike count of an example neuron from each model [receptive field in ***A***; dots: mean, error bars: standard deviation across models (n=20)] ***D***, PID values between neuron spike counts and the location of the stimulus for pairs of neurons as a function of the distance between the centers of the receptive fields of the neurons. [For all information plots, dots: mean, error bars: standard deviation across models (n=20)]. Note that redundancy was maximized for overlapping receptive fields, unique information peaked for neighboring place fields, and synergy peaked for concentric receptive fields. Furthermore, synergy values were substantially higher than redundancy indicating that synergy dominates joint encoding in this system.

To model this system, we randomly placed 300 neurons in a two-dimensional square plane (1 unit by 1 unit). Periodic boundary conditions were used to remove edge effects. An example neuron’s receptive field is shown in Fig. 20*A* along with three example stimuli in Fig. 20*B*. By calculating the mutual information between a neuron’s spike count in 25-ms bins and the location of the stimuli (discretized by dividing the plane into 16 equal sized squares), we found that the neurons encoded the location of the stimuli (Fig. 20*C*).

Next, we applied the partial information decomposition analysis with the spike count of pairs of neurons as the X variables and the location of the light stimulus as the Y variable (Fig. 20*D*,*E*). As expected, when the two cells’ receptive fields were located near each other, high redundancy and low unique information were present. This is expected, because pairs of neurons close together would exhibit similar responses to stimuli. Conversely, lower redundancy and higher unique information were observed when the cells’ receptive fields were far apart (Fig. 20*D*). When the cells’ receptive fields were far apart, they exhibited different responses to stimuli, so they provided more unique information about the stimuli locations. As a result, the redundancy decreased (recall that the mutual information for each neuron alone about the stimulus location was relatively constant; see Eqn. 21). The synergy between neuron pairs was highest when the cells’ receptive fields were close together (Fig. 20*D*). Also, note that the magnitude of the synergy was much higher than the magnitude of the redundancy. This indicates that two neurons always provide a large boost in encoding (synergy) when considered together relative to being considered alone regardless of their relative positions. In this example, information theory identified and quantified stimulus encoding by the neurons and how encoding performed by pairs of neurons interacts with the physical separation between their receptive fields.

### Movement direction and motor cortex neurons

The third canonical neuroscience system we examined was movement direction encoding by motor cortex neurons (Georgopoulos et al., 1982; Bear et al., 2007). In a relatively simple task, a monkey is trained to move a cursor from a central hold position to one of eight target positions circularly arranged around the center position. It has been shown (Georgopoulos et al., 1982; Bear et al., 2007) that some neurons in the primary motor cortex exhibit increased or decreased firing shortly before the monkey begins moving the cursor based on the desired direction of motion. Cells exhibit certain preferred directions for which their firing will increase. Movements opposite to the preferred direction cause decreased firing.

**Figure 21.**
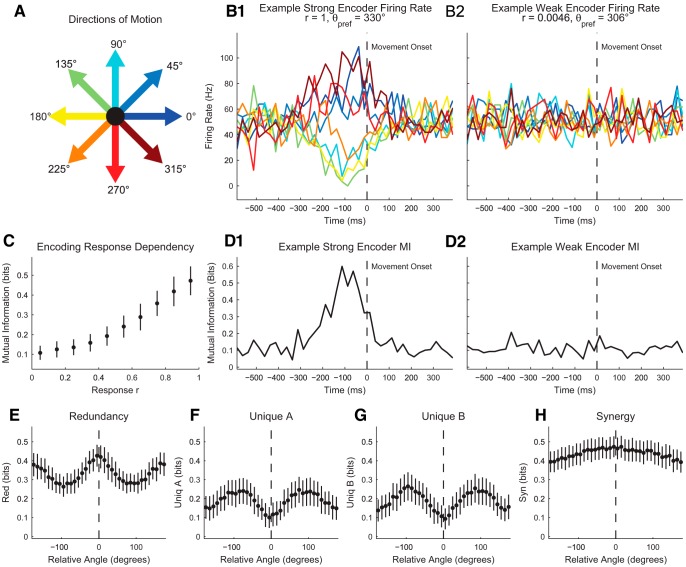
Model primary motor cortex neurons jointly encode movement direction. ***A***, Possible directions of motion. ***B***, Example firing rate profiles for a strong direction encoder (B1) and a weak direction encoder (B2). ***C***, Maximum mutual information between the direction of motion and the spike count of a neuron as a function of the strength of neuron response to direction. ***D***, Example mutual information between the direction of motion and the spike count of the neuron for the corresponding examples from (B). ***E–H***, PID values between the spike count of pairs of neurons and the direction of motion as a function of the difference in preferred firing angle between the neurons for only strong encoders (r=1). Note, elevated redundancy was observed for parallel and antiparallel preferred firing angles, while elevated unique information was observed for perpendicular preferred firing angles. Synergy was relatively constant for all angle differences. [For all information plots, dots: mean, error bars: standard deviation across models (n=20)].

To simulate the behavior of these cells, we used a similar model task where an animal was required to move a cursor in one of eight randomly chosen directions (Fig. 21*A*). We used model cells that increased or decreased (with varying degrees of responsiveness, r) their firing based on their randomly assigned preferred direction of motion and the actual direction of motion (Fig. 21*B*). As expected, highly responsive neurons showed high mutual information between the direction of motion and the spike count of the neuron in 25-ms bins, while unresponsive neurons did not (Fig. 21*C*,*D*).

Next, we applied the partial information decomposition analysis with the spiking activity of pairs of neurons (sorted using relative preferred direction) as the X variables and the location of the direction of motion as the Y variable (Fig. 21*E–H*). High redundancy was observed when the preferred directions for the neurons were parallel or antiparallel (Fig. 21*E*), while high unique information was observed when the preferred directions were perpendicular (Fig. 21*F*,*G*). This result is intuitive because neurons with parallel or antiparallel preferred directions best encode the same directions of motion. This is because neurons can best distinguish movements in their preferred direction from the opposite direction. However, neurons with perpendicular preferred directions best encode different movement directions. High synergy was observed at all relative angles (Fig. 21*H*). Thus, pairs of direction encoders provided a sizable boost (synergy) when considered together beyond their individual encoding. In this example, information theory identified direction encoding and how the encoding performed by pairs of neurons interacts with neuron preferred firing direction.

### Place cells

For our last canonical experiment, we examined a simulation of hippocampal place cells (O’Keefe and Dostrovsky, 1971; Bear et al., 2007). These neurons preferentially fire when an animal is located at a certain point in an environment (so-called “place field”).

**Figure 22.**
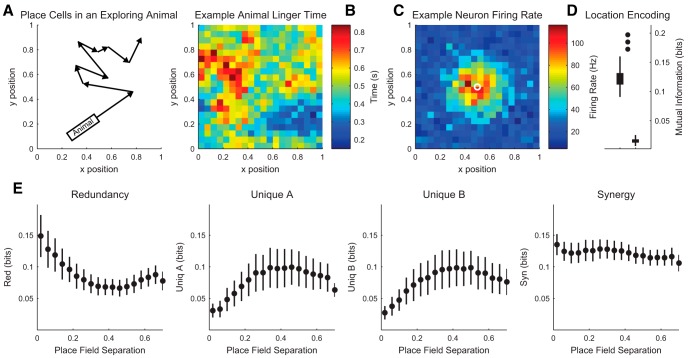
Joint encoding by model place cells is distance dependent. ***A***, A model animal was allowed to randomly walk on a 2-D surface with periodic boundary conditions. ***B***, Example animal linger time as a function of position. ***C***, An example place cell shows elevated firing when the animal was near its place field (white circle). ***D***, Place cells encoded the location of the animal better than nonplace cells that did not respond to location. (Thin bars: min to max range, thick bars: interquartile range, rank-sum test, *p* < 0.001.) ***E***, PID values between neuron spike counts and the location of the animal for pairs of neurons as a function of the distance between the centers of the place fields of the neurons. [For all information plots, dots: mean, error bars: standard deviation across models (n=20)]. Note that redundancy was maximized for overlapping place fields, unique information peaked for neighboring place fields, and synergy was elevated regardless of the relative positions of the neurons.

To simulate these cells, we used a model animal that performed a random walk through a 2-dimensional plane with periodic boundary conditions to remove edge effects (Fig. 22*A*). Based on the random walk, the animal spent more time in certain parts of the environment than others (Fig. 22*B*). Each place cell was randomly assigned a certain location (place field) for which it would fire preferentially (Fig. 22*C*). By calculating the mutual information between the spike count of a neuron in 100-ms bins and location of the animal (discretized by dividing the plane into 16 equal-sized squares), we found that place cells encoded more information about the location of the animal than cells that did not preferentially fire based on the location of the animal (Fig. 22*D*).

Finally, we applied the partial information decomposition analysis with the spiking activity of pairs of neurons as the X variables and the location of the animal as the Y variable (Fig. 22*E*). As we might expect, when the place fields for both neurons were close together, high redundancy and low unique information resulted. However, when the place fields were far apart, the redundancy decreased and the unique information increased. The synergy between the neurons was relatively high regardless of the relative location of the neurons. In this example, information theory identified place cells and how the encoding performed by pairs of neurons interacts with neuron place fields.

## Discussion

### Key points

In this article, we reviewed basic information theory measures and the logistics of applying those measures to data generated by neuroscience experiments. We examined example analyses of 13 simulations of neural spiking data using the freely available Matlab Neuroscience Information Theory Toolbox. These demonstrations highlighted several noteworthy features of information theory analyses. Mutual information can be used to measure the encoding of stimulus and behavioral information by individual neurons. Transfer entropy and information transmission can be used to measure information flow between neurons. The partial information decomposition can be used to break down encoding by two variables into redundant, unique, and synergistic parts. Finally, the precise interpretation of an information theory analysis is dependent on the assignment of variables (e.g., time delay, bin size, converging or diverging schemes with the partial information decomposition, etc.).

### Alternative methods and limitations

As we discussed in above, information theory possesses several distinct advantages over alternative analysis methods. Information theory is model independent, it can be applied to any mixture of data types, it is capable of detecting linear and nonlinear interactions, it is multivariate, and it produces results in general units, which facilitates size effect comparisons.

We would like to emphasize something quite remarkable about information theory. Nowhere in our definitions did we assume any particular structure to the data. We did not assume the data were linearly related or that the data followed some canonical distribution (e.g., normal, Poisson, or exponential), unlike correlation, Bayesian analyses, *t* tests, and many other measures of interactions between variables. We are not fitting the data to some model. The model-free nature of information theory analysis makes it a flexible tool, which is especially valuable when the underlying rules governing the system are not known.

While the various features of information theory are certainly advantageous in many circumstances, limitations to information theory analyses do exist and other methods may be preferable in certain scenarios. For instance, in their most basic form discussed in this introductory article, information theory analyses require discretized data. Thus, continuous data must be discretized (see *Probability Distributions and Initial Analysis Steps*), which requires assumptions about the number of bins and/or bin size and which can induce bias and affect the results. In other words, using three bins instead of four will produce different results, especially in terms of information values (see Daw et al. (2003) for a review of discretization methods and their impacts on data analyses from a more general perspective). Several more advanced methods have been developed, some of which are implemented in other software packages, to overcome these problems in certain cases (Nemenman et al., 2002; Goldberg et al., 2009; Ince et al., 2009; Magri et al., 2009; Ito et al., 2011; Lindner et al., 2011; Pastore et al., 2016). Furthermore, even with the basic analysis methods presented herein, statistical testing methods exist to reduce the appearance of false-positive information theory results caused by discretization and bias effects (see *Significance Testing*). Still, these issues must be considered when choosing the appropriate analysis technique.

In connection with issues surrounding data discretization, the amount of data required to perform an information theory analysis can be large, especially in systems with many discrete states and/or many variables. For instance, in a system with three variables that each have four discrete states, there will be 64 unique joint states, requiring at least (but preferably much more than) 64 joint observations to analyze. Depending on the experiment, it may be possible to gather this many observations or it may be possible to analyze variables with fewer discrete states. Statistical testing methods will reduce the appearance of false-positive results based on too little data, but this feature of information theory analyses must be considered because it can limit the ability of the analysis to detect true-positives.

It goes without saying that there are numerous alternative methods for analyzing neuroscience data. Basic correlation measures are used throughout neuroscience and science in general, though these methods often assume the underlying data are linear, so these methods are not model independent. Cross-correlation is widely used in the analysis of neural spiking data (Bonifazi et al., 2009; Ito et al., 2014) to assess functional connections between pairs of neurons (i.e., neuron A tends to spike just before neuron B spikes). These analyses are similar in design to transfer entropy analyses (Ito et al., 2011; Timme et al., 2014b). However, cross-correlation is model dependent, unlike transfer entropy. Granger causality has been widely used to assess causal interactions between both continuous and discrete neural signals (Granger, 1969; Ding et al., 2006; Nakhnikian et al., 2014), though underlying assumptions about the analysis (e.g., Gaussian distributed data) must be thoroughly evaluated. Additionally, neural encoding is frequently assessed by applying statistical tests to neural signal observations under two conditions (e.g., stimulus on versus off or behavior A versus B). Roughly speaking, this method is akin to saying a neuron encodes X if the neuron spikes a lot for X, but spikes very little for not X. This method is relatively straightforward, but it requires a system with only two states (e.g., stimulus on and off), it is difficult to compare across systems or neurons (i.e., does the neuron encode stimulus A better than stimulus B?), and it requires the selection of the correct statistical test given the distribution of the neural data. An information theory analysis of such a system does not have these requirements. Finally, multivariate interactions, which can be analyzed with the partial information decomposition, are difficult to analyze with linear methods.

We wish to emphasize that while these other model-dependent methods lack the flexibility of information theory because it is model independent, the model-dependent methods are frequently able to leverage the assumptions tied to the underlying model to achieve greater power with less data. Additionally, linear analysis methods typically require far less computation time. Therefore, linear or model-dependent methods can be very useful in analyses of neural data, and frequently it is helpful to start an analysis with a simple linear method.

As we discussed in above, it is important to emphasize that information theory analyses do not produce models that describe how the data were generated. Because the creation of such models is a primary goal of science, it is important to note that information theory analyses can be used as powerful guides to building complex models that can describe neuroscience data (though see James and Crutchfield (2017) for further discussion). We believe that Bayesian analyses represent the preferred method for building such models (Friston et al., 2003; Koller and Friedman, 2009; Kruschke, 2015), though many other model-fitting procedures (i.e., regression analyses) exist (Zar, 2010). Of course, a researcher may choose to simply apply some type of model fitting without first applying an information theory analysis, but doing so leaves open the possibility that some type of interaction exists in the data that is not captured by the model. Each researcher should careful weigh whether this is a relevant concern for his or her experimental questions.

### Possible neuroscience applications

While the demonstrations we employed throughout this introductory tutorial were focused on neural spiking, clearly many other types of data are used widely in neuroscience. We chose to focus on neural spiking data due to our expertise with it. However, we wish to emphasize that nearly identical analyses could easily be performed with BOLD signal data from fMRI studies, fluorescence data from calcium imaging studies, or voltage signals from extracellular, EEG, or MEG studies. Certainly, sampling constraints (e.g., slower time resolution in fMRI and calcium imaging) or other pre-processing steps (e.g., initial power spectrum decomposition in EEG) would alter the results of the analyses or the precise details of how they were applied, but the distribution of voltage values, BOLD signals, or fluorescence signals across trials or time can just as easily be discretized as spike counts using the methods discussed above (see *Probability Distributions and Initial Analysis Steps*).

Indeed, even other types of data that are conceptually different from the various measures of neural activity discussed above can be easily treated with these information theory tools. Information theory is currently used in genetics (Vinga, 2013; Ignac et al., 2014; Smouse et al., 2015), but studies could be performed linking genetics and neuroscience. For instance, it is possible to examine how neural activity, neural responses to stimuli, or animal behavior relate to genetic information by examining model animals with certain genetic differences. Furthermore, using the partial information decomposition, it is possible to ask whether certain genes work synergistically, uniquely, or redundantly to predict certain effects in organisms. In short, the limiting factor in information theory analyses is not the information theory analysis itself, it is the researcher’s ability to gather the right type of data to address his or her experimental question.

## Synthesis

Reviewing Editor: Marlene Bartos, University of Freiburg Decisions are customarily a result of the Reviewing Editor and the peer reviewers coming together and discussing their recommendations until a consensus is reached. When revisions are invited, a fact-based synthesis statement explaining their decision and outlining what is needed to prepare a revision will be listed below. The following reviewer(s) agreed to reveal their identity: Nicholas Hatsopoulos, Ryan James.

The study was reviewed by two experts in the field of information theory. Both valued the manuscript as a useful introduction to information theory as it applies to neuroscience experiments using spiking data. It is very well written and was designed for someone with basic understanding of probability theory and statistics. The manuscript also provided some useful neuroscience examples to clarify the usefulness of information theory and provides software, from which the analysis can be reproduced.

Reviewer 1

Criticisms

1. The discussion of the “uniform count” binning method deserves a bit more explanation. It was unclear to me why it is more powerful than “equal width” binning and how it can be used as a single null model for significance testing.

2. Perhaps I missed something fundamental, but in the examples such as 5.1, it was unclear to me why information in the spike counts about the stimulus (on or off) is high only when the stimulus is on and zero when it is off. Doesn'9;t a lack of spikes also provide information that the stimulus is off.

Minor typos:

1. On line 203, “feed” should be “fed”.

2. On line 353, “base” should be “based”.

Reviewer 2

1. It is repeatedly suggested that an information-theoretic analysis can be used to inform a modeler at to which components of a system are interacting, and which are not. This is perhaps optimistic, see `this article <http://www.mdpi.com/1099-4300/19/10/531>`_ for an example and discussion.

2. It is frequently mentioned that information theory allows for multivariate analysis, and yet there is very little discussion of the myriad of multivariate measures that exist. This is perhaps a strength, in that those measures generally lack operational meaning, and only exist and multivariate generalizations of well-founded uni- or bivariate information measures. In any case, it is mildly misleading to hype the multivariate nature of information theory and then only discuss joint entropy, standard mutual information with vector-valued variables, conditional mutual information, and the partial information decomposition.

Specific Issues

---------------

1. Near line 200, it is claimed that information theory can be utilized to determine causality. This is, at best, a controversial opinion. It can be said that certain information-theoretic measures (`transfer entropy <http://eprints.cs.univie.ac.at/4968/>`_, `directed information <http://www.mdpi.com/1099-4300/15/1/113/htm>`_, `causation entropy <https://www.sciencedirect.com/science/article/pii/S0167278913001991>`_) subsume Granger causality, and there are other measures attempting to make Pearl's do-calculus information-theoretic (see `here <http://www.worldscientific.com/doi/abs/10.1142/S0219525908001465>`_.

2. Toward the end of section 4.2.1 the differences between discrete and continuous information theory are briefly discussed. It is my opinion that an important aspect of this difference is not mentioned: namely, that they are beasts of a different nature. Primarily, differential entropy can be negative, and this makes it obvious that the discrete entropy of a binned continuous variable, in the limit of infinitely fine binning, does not limit to the differential entropy. While I understand that this oddity is left out to simplify matters, I would find a footnote mentioning this issue to be appropriate.

3. Though it is said that different choices of binning can drastically affect the results of an information-theoretic analysis, no rational for this is given. Perhaps a brief footnote pointing to one of the multitude of papers discussing the impact of non-generating partitions on the symbolic dynamics of a dynamical system would be appropriate.

4. In section 4.3, you should include the dependent coins distribution as an example. Its entropy is 1.721928094887362 and the conditional entropy of either coin given the other is 0.721928094887362.

5. On line 724 it is claimed that the conditional mutual information quantifies the interactions between two variables “after removing the effects of a third variable.” This is untrue and highly misleading: were that the case, the inequality I[X:Y|Z] <= I[X:Y] would hold. As a trivial example, consider two independent fair coins valued 0 and 1, X and Y, and let Z = X xor Y. Each pair of variables is independent, I[X:Y] = 0. Yet I[X:Y|Z] = 1, because Z determines if X and Y are identical or opposite. It absolutely can not be said that this conditioning is “removing” the effects of Z. A better colloquial description is that conditioning “takes into account” the effects of a third variable, which can either reduce the interactions --- conditional independence --- or enhance it --- conditional dependence.

6. In section 4.11 many different information theory packages are discussed. It might be prudent to include the package `dit <https://github.com/dit/dit>`_. Although it lacks many useful tools for working with empirical data, it is likely the most comprehensive package available when it comes to information measures.

Conclusion

----------

Overall I find the work to be well written and an important introduction of information theory to neuroscience, a field which could broadly benefit from its application. As stated above, I do have some concerns as to how some aspects are presented. Importantly, specific point 5 above. This common misunderstanding needs to addressed, and this work should not perpetuate such a damaging misinterpretation of the measure. Once the points above have been addressed or refuted, I would recommend it for publication.

## References

[B1] Adriaans P (2012) Information In: Standford Encyclopedia of Philosophy (Zalta EN, ed.).

[B2] Asaad WF, Lauro PM, Perge JA, Eskandar EN (2017) Prefrontal neurons encode a solution to the credit-assignment problem. J Neurosci 37:6995–7007. 10.1523/JNEUROSCI.3311-16.2017 28634307 PMC5518425

[B3] Bear MF, Connors BW, Paradiso MA (2007) Neuroscience: exploring the brain, Third Edition Baltimore, MD: Lippincott Williams and Wilkins.

[B4] Beer RD, Williams PL (2014) Information processing and dynamics in minimally cognitive agents. Cogn Sci 1–38. 10.1111/cogs.12142 25039535

[B5] Benjamini Y, Hochberg Y (1995) Controlling the false discovery rate: a practical and powerful approach to multiple testing. J R Stat Soc B 57:289–300.

[B6] Benjamini Y, Yekutieli D (2001) The control of the false discovery rate in multiple testing under dependency. Ann Stat 29:1165–1188. 10.1214/aos/1013699998

[B7] Bertschinger N, Rauh J, Olbrich E, Jost J, Ay N (2014) Quantifying unique information. Entropy 16:2161–2183. 10.3390/e16042161

[B8] Bettencourt LMA, Gintautas V, Ham MI (2008) Identification of functional information subgraphs in complex networks. Phys Rev Lett 100:238701. 10.1103/PhysRevLett.100.238701 18643550

[B9] Bialek W, Rieke F, De Ruyter van Steveninck RR, Warland D (1991) Reading a neural code. Science 252:1854–1857. 10.1126/science.2063199 2063199

[B10] Bonachela JA, Hinrichsen H, Muñoz MA (2008) Entropy estimates of small data sets. J Phys A 41:202001. 10.1088/1751-8113/41/20/202001

[B11] Bonifazi P, Goldin M, Picardo MA, Jorquera I, Cattani A, Bianconi G, Represa A, Ben-Ari Y, Cossart R (2009) GABAergic hub neurons orchestrate synchrony in developing hippocampal networks. Science 326:1419–1424. 10.1126/science.1175509 19965761

[B12] Borst A, Theunissen FE (1999) Information theory and neural coding. Nat Neurosci 2:947–957. 10.1038/14731 10526332

[B13] Bossomaier T, Barnett L, Harre M, Lizier JT (2016) An introduction to transfer entropy. New York: Springer International.

[B14] Brenner N, Strong SP, Koberle R, Bialek W, de R, van Steveninck RR (2000) Synergy in a neural code. Neur Comput 12:1531–1532. 10.1162/089976600300015259 10935917

[B15] Bullmore E, Sporns O (2009) Complex brain networks: graph theoretical analysis of structural and function systems. Nat Rev Neurosci 10:186–198. 10.1038/nrn2618 19190637

[B16] Butts DA (2003) How much information is associated with a particular stimulus? Netw Comput Neur Syst 14:177–187. 10.1088/0954-898X_14_2_301 12790180

[B17] Butts DA, Weng C, Jin J, Yeh C, Lesica NA, Alonso J, Stanley GB (2007) Temporal precision in the neural code and the timescales of natural vision. Nat Lett 449:92–95. 10.1038/nature06105 17805296

[B18] Castellucci V, Pinsker H, Kupfermann I, Kandel ER (1970) Neuronal mechanisms of habituation and dishabituation of the gill-withdrawal reflex in aplysia. Science 167:1745–1748. 5416543 10.1126/science.167.3926.1745

[B19] Cover TM, Thomas JA (2006) Elements of Information Theory, 2nd Edition New York: Wiley-Interscience.

[B20] Cunningham JP, Yu BM (2014) Dimensionality reduction for large-scale neural recordings. Nat Neurosci 17:1500–1509. 10.1038/nn.3776 25151264 PMC4433019

[B21] Daw CS, Finney CEA, Tracy ER (2003) A review of symbolic analysis of experimental data. Rev Scientif Instr 74:915–930. 10.1063/1.1531823

[B22] DeWeese MR, Meister M (1999) How to measure the information gained from one symbol. Netw Comput Neur Syst 10:325–340. 10.1088/0954-898X_10_4_303 10695762

[B23] Dimitrov AG, Lazar AA, Victor JD (2011) Information theory in neuroscience. J Comput Neurosci 30:1–5. 10.1007/s10827-011-0314-3 21279429 PMC3736735

[B24] Ding M, Chen Y, Bressler SL (2006) Granger causality: basic theory and application to neuroscience In: Handbook of Time Series Analysis: Recent Theoretical Developments and Applications (Schelter B, Winterhalder M, Timmer J, eds), *p* 437 Weinheim, Germany: Wiley-VCH.

[B25] Dit-Contributors (2018) Dit: Discrete information theory. Available at https://dit.readthedocs.io/en/latest/.

[B26] Finn C, Lizier JT (2018) Pointwise information decomposition using the specificity and ambiguity lattices. arXiv doi:10.3390/ecea-4-05024. 10.3390/e20040297 PMC7512814 33265388

[B27] Friston KJ (2011) Functional and effective connectivity: a review. Brain Connect 1:13–36. 10.1089/brain.2011.0008 22432952

[B28] Friston KJ, Harrison L, Penny W (2003) Dynamic causal modelling. Neuroimage 19:1273–1302. 10.1016/S1053-8119(03)00202-7 12948688

[B29] Georgopoulos AP, Kalaska JF, Caminiti R, Massey JT (1982) On the relations between the direction of two-dimensional arm movements and cell discharge in primate motor cortex. J Neurosci 2:1527–1537. 7143039 10.1523/JNEUROSCI.02-11-01527.1982 PMC6564361

[B30] Goldberg DH, Victor JD, Gardner EP, Gardner D (2009) Spike train analysis toolkit: enabling wider application of information-theoretic techniques in neurophysiology. Neuroinformatics 7:165–178. 10.1007/s12021-009-9049-y 19475519 PMC2818590

[B31] Gomez-Herraro G, Wu W, Rutanen K, Soriano MC, Pipa G, Vicente R (2015) Assessing coupling dynamics from an ensemble of time series. Entropy 17:1958–1970. 10.3390/e17041958

[B32] Granger CWJ (1969) Investigating causal relations by econometric models and cross-spectral methods. Econometrica 37:424–438. 10.2307/1912791

[B33] Griffith V, Chong EKP, James RG, Ellison CJ, Crutchfield JP (2014) Intersection information based on common randomness. Entropy 16:1985–2000. 10.3390/e16041985

[B34] Harder M, Salge C, Polani D (2013) Bivariate measure of redundant information. Phys Rev E 87:012130. 10.1103/PhysRevE.87.012130 23410306

[B35] Honey CJ, Kotter R, Breakspear M, Sporns O (2007) Network structure of cerebral cortex shapes functional connectivity on multiple time scales. Proc Natl Acad Sci U S A 104:10240–10245. 10.1073/pnas.0701519104 17548818 PMC1891224

[B36] Hramov AE, Koronovskii AA, Makarov VA, Pavlov AN, Sitnikova E (2015) Wavelets in neuroscience. Berlin: Springer.

[B37] Ignac TM, Skupin A, Sakhanenko NA, Galas DJ (2014) Discovering pair-wise genetic interactions: an information theory-based approach. PLOS One 9:e92310. 10.1371/journal.pone.0092310 24670935 PMC3966778

[B38] Ince RAA (2017) Measuring multivariate redundant information with pointwise common change in surprisal. Entropy 19:318–344. 10.3390/e19070318

[B39] Ince RAA, Petersen RS, Swan DC, Panzeri S (2009) Python for information theoretic analysis of neural data. Front Neuroinform 3:4. 10.3389/neuro.11.004.2009 19242557 PMC2647335

[B40] Ince RAA, Mazzoni A, Petersen RS, Panzeri S (2010) Open source tools for the information theoretic analysis of neural data. Front Neurosci 4:62–70. 20730105 10.3389/neuro.01.011.2010 PMC2891486

[B41] Ince RAA, Rijsbergen NJ, Thut G, Rousselet GA, Gross J, Panzeri S, Schyns PG (2015) Tracing the flow of perceptual features in an algorithmic brain network. Sci Reports 5:17681. 10.1038/srep17681 PMC4669501 26635299

[B42] Ito S, Hansen ME, Heiland R, Lumsdaine A, Litke AM, Beggs JM (2011) Extending transfer entropy improves identification of effective connectivity in a spiking cortical network model. PloS One 6:e27431. 10.1371/journal.pone.0027431 22102894 PMC3216957

[B43] Ito S, Yeh FC, Hiolski E, Rydygier P, Gunning DE, Hottowy P, Timme N, Litke AM, Beggs JM (2014) Large-scale, high-resolution multielectrode-array recording depicts functional network differences of cortical and hippocampal cultures. PloS One 9:e105324. 10.1371/journal.pone.0105324 25126851 PMC4134292

[B44] Izhikevich EM (2003) Simple model of spiking neurons. IEEE Trans Neur Netw 14:1569–1572. 10.1109/TNN.2003.820440 18244602

[B45] Izhikevich EM (2007) Dynamical Systems in Neuroscience. Cambridge, MA: MIT Press.

[B46] James RG, Crutchfield JP (2017) Multivariate dependence beyond Shannon information. Entropy 19:531–546. 10.3390/e19100531

[B47] Jeong J, Gore JC, Peterson BS (2001) Mutual information analysis of the EEG in patients with Alzheimer’s disease. Clin Neurophysiol 112:827–835. 11336898 10.1016/s1388-2457(01)00513-2

[B48] Koller D, Friedman N (2009) Probabilistic Graphical Models: Principles and Techniques. Cambridge, MA: MIT Press.

[B49] Kraskov A, Stoegbauer H, Grassberger P (2004) Estimating mutual information. Phys Rev E 69:066138. 10.1103/PhysRevE.69.066138 15244698

[B50] Kruschke JK (2015) Doing Bayesian Data Analysis. London: Elsevier.

[B51] Kuffler SW (1953) Discharge patterns and functional organization of mammalian retina. J Neurophysiol 16:37–68. 10.1152/jn.1953.16.1.37 13035466

[B52] Lindner M, Vicente R, Priesemann V, Wibral M (2011) TRENTOOL: a MATLAB open source toolbox to analyse information flow in time series data with transfer entropy. BMC Neurosci 12:119. 10.1186/1471-2202-12-119 22098775 PMC3287134

[B53] Lizier JT (2014) JIDT: an information-theoretic toolkit for studying the dynamics of complex systems. Front Robotics AI 1:11. 10.3389/frobt.2014.00011

[B54] Lizier JT, Prokopenko M, Zomaya AY (2008) Local information transfer as a spatiotemporal filter for complex systems. Phys Rev E 77:026110. 10.1103/PhysRevE.77.026110 18352093

[B55] Lizier JT, Heinzle J, Horstmann A, Haynes J, Prokopenko M (2011) Multivariate information-theoretic measures reveal directed information structure and task relevant changes in fMRI connectivity. J Comput Neurosci 30:85–107. 10.1007/s10827-010-0271-2 20799057

[B56] Magri C, Whittingstall K, Singh V, Logothetis NK, Panzeri S (2009) A toolbox for the fast information analysis of multiple-site LFP, EEG, and spike train recordings. BMC Neurosci 10:81. 10.1186/1471-2202-10-81 19607698 PMC2723115

[B57] McShane BB, Gal D, Gelman A, Robert C, Tackett JL (2017) Abandon statistical significance. arXiv 1709.07588.

[B58] Montalto A, Faes L, Marinazzo D (2014) MuTE: a MATLAB toolbox to compare established and novel estimators of the multivariate transfer entropy. PloS One 9:e109462. 10.1371/journal.pone.0109462 25314003 PMC4196918

[B59] Moore DG, Valentini G, Walker SI, Levin M (2017) Inform: a toolkit for information-theoretic analysis of complex systems. In 2017 IEEE Symposium Series on Computational Intelligence (SSCI).

[B60] Myung IJ (2003) Tutorial on maximum likelihood estimation. J Math Psychol 47:90–100. 10.1016/S0022-2496(02)00028-7

[B61] Nakhnikian A, Rebec GV, Grasse LM, Dwiel LL, Shimono M, Beggs JM (2014) Behavior modulates effective connectivity between cortex and striatum. PloS One 9:e89443. 10.1371/journal.pone.0089443 24618981 PMC3949668

[B62] Nemenman I, Shafee F, Bialek W (2002) Entropy and inference, revisited. Advances in Neural Information Processing Systems. Cambridge, MA: MIT Press 471–478.

[B63] Nemenman I, Bialek W, De Ruyter van Steveninck RR (2004) Entropy and information in neural spike trains: progress on the sampling problem. Phys Rev E 69:056111. 10.1103/PhysRevE.69.056111 15244887

[B64] Nigam S, Shimono M, Ito S, Yeh FC, Timme N, Myroshnychenko M, Lapish CC, Tosi Z, Hottowy P, Smith WC, Masmanidis SC, Litke AM, Sporns O, Beggs JM (2016) Rich-club organization in effective connectivity among cortical neurons. J Neurosci 36:670–684. 10.1523/JNEUROSCI.2177-15.2016 26791200 PMC4719009

[B65] O’Keefe J, Dostrovsky J (1971) The hippocampus as a spatial map. Brain Res 34:171–175. 5124915 10.1016/0006-8993(71)90358-1

[B66] Paninski L (2003) Estimation of entropy and mutual information. Neur Comput 15:1191–1253. 10.1162/089976603321780272

[B67] Panzeri S, Treves A (1996) Analytical estimates of limited sampling biases in different information measures. Netw Comput Neur Syst 7:87–107. 10.1080/0954898X.1996.11978656 29480146

[B68] Panzeri S, Schultz SR (2001) A unified approach to the study of temporal, correlational, and rate coding. Neur Comput 13:1311–1349. 11387048 10.1162/08997660152002870

[B69] Panzeri S, Senatore R, Montemurro MA, Petersen RS (2007) Correcting for the sampling bias problem in spike train information measures. J Neurophysiol 98:1064–1072. 10.1152/jn.00559.2007 17615128

[B70] Panzeri S, Petersen RS, Schultz SR, Lebedev M, Diamon ME (2001) The role of spike timing in the coding of stimulus location in rat somatosensory cortex. Neuron 29:769. 11301035 10.1016/s0896-6273(01)00251-3

[B71] Pastore VP, Poli D, Godjoski A, Martinoia S, Massobrio P (2016) ToolConnect: a functional connectivity toolbox for in vitro networks. Front Neuroinform 10:13. 10.3389/fninf.2016.00013 27065841 PMC4811958

[B72] Pica G, Piasini E, Chicharro D, Panzeri S (2017) Invariant components of synergy, redundancy, and unique information among three variables. arXiv 1706.08921.

[B73] Quax R, Har-Shemesh O, Sloot PMA (2017) Quantifying synergistic information using intermediate stochastic variables. Entropy 19:85. 10.3390/e19020085

[B74] Quinn CJ, Coleman TP, Kiyavash N, Hatsopoulos N (2011) Estimating the directed information to infer causal relationships in ensemble neural spike train recordings. J Comput Neurosci 30:17–44. 10.1007/s10827-010-0247-2 20582566 PMC3171872

[B75] Quiroga RQ, Panzeri S (2009) Extracting information from neuronal populations: information theory and decoding approaches. Nat Rev Neurosci 10:173–185. 10.1038/nrn2578 19229240

[B76] Quiroga RQ, Panzeri S, eds (2013) Principles of Neural Coding. Boca Raton, FL: CRC Press.

[B77] Ramos AMT, Macau EEN (2017) Minimum sample size for reliable causal inference using transfer entropy. Entropy 19:150. 10.3390/e19040150

[B78] www.nicholastimme.com (2018) Redacted for double-blind review.

[B79] Rieke F, Warland D, de Ruyter van Steveninck RR, Bialek W (1997) Spikes: Exploring the Neural Code. Cambridge, MA: MIT Press.

[B80] Rolston JD, Wagenaar DA, Potter SM (2007) Precisely timed spatiotemporal patterns of neural activity in dissociated cortical cultures. Neuroscience 148:294–303. 10.1016/j.neuroscience.2007.05.025 17614210 PMC2096414

[B81] Schneidman E, Bialek W, Berry MJ, II (2003a) Synergy, redundancy, and independence in population codes. J Neurosci 23:11539–11553. 10.1523/JNEUROSCI.23-37-11539.2003 14684857 PMC6740962

[B82] Schneidman E, Still S, Berry MJ, II, Bialek W (2003b) Network information and connected correlations. Phys Rev Lett 91:238701. 10.1103/PhysRevLett.91.238701 14683220

[B83] Schölvinck ML, Leopold DA, Brookes MJ, Khader PH (2013) The contribution of electrophysiology to functional connectivity mapping. NeuroImage 80:297–306. 10.1016/j.neuroimage.2013.04.010 23587686 PMC4206447

[B84] Schreiber T (2000) Measuring information transfer. Phys Rev Lett 85:461–464. 10.1103/PhysRevLett.85.461 10991308

[B85] Shannon CE (1948) A mathematical theory of communication. Bell Sys Tech J 27:379–423. 10.1002/j.1538-7305.1948.tb01338.x

[B86] Smouse PE, Whitehead MR, Peakall R (2015) An informational diversity framework, illustrated with sexually deceptive orchids in early stages of speciation. Mol Ecol Res 15:1375–1384. 10.1111/1755-0998.12422 25916981

[B87] Sporns O (2007) Brain connectivity. Scholarpedia 2:4695. 10.4249/scholarpedia.4695

[B88] Sporns O (2013) Structure and function of complex brain networks. Dialog Clin Neurosci 15:247–262. 24174898 10.31887/DCNS.2013.15.3/osporns PMC3811098

[B89] Staniek M, Lehnertz K (2008) Symbolic transfer entropy. Phys Rev Lett 100:158101. 10.1103/PhysRevLett.100.158101 18518155

[B90] Stanley GB (2013) Reading and writing the neural code. Nat Neurosci 16:259–263. 10.1038/nn.3330 23434978

[B91] Stone JV (2018) Information theory: a tutorial introduction. arXiv 1802.05968.

[B92] Szabo Z (2014) Information theoretical estimators toolbox. J Mach Learn Res 15:283–287.

[B93] Timme N, Alford W, Flecker B, Beggs JM (2014a) Synergy, redundancy, and multivariate information measures: an experimentalist’s perspective. J Comput Neurosci 36:119–140. 10.1007/s10827-013-0458-4 23820856

[B94] Timme N, Ito S, Myroshnychenko M, Yeh FC, Hiolski E, Hottowy P, Beggs JM (2014b) Multiplex networks of cortical and hippocampal neurons revealed at different timescales. PLoS One 9:e115764. 10.1371/journal.pone.0115764 25536059 PMC4275261

[B95] Timme NM, Ito S, Myroshnychenko M, Nigam S, Shimono M, Yeh FC, Hottowy P, Litke A, Beggs JM (2016) High-degree neurons feed cortical computations. PLoS Comput Biol 12:e1004858. 10.1371/journal.pcbi.1004858 27159884 PMC4861348

[B112] Timme NM, Lapish C (2018) GitHub - nmtimme/Neuroscience-Information-Theory-Toolbox: A MATLAB toolbox for performing information theory analyses of neuroscience data. https://github.com/nmtimme/Neuroscience-Information-Theory-Toolbox

[B96] Treves A, Panzeri S (1995) The upward bias in measures of information derived from limited data samples. Neur Comput 7:399–407. 10.1162/neco.1995.7.2.399

[B97] Van Rullen R, Thorpe SJ (2001) Rate coding versus temporal order coding: what the retinal ganglion cells tell the visual cortex. Neur Comput 13:1255–1283. 11387046 10.1162/08997660152002852

[B98] Vicente R, Wibral M, Lindner M, Pipa G (2011) Transfer entropy: a model-free measure of effective connectivity for the neurosciences. J Comput Neurosci 30:45–67. 10.1007/s10827-010-0262-3 20706781 PMC3040354

[B99] Victor JD (2002) Binless strategies for estimation of information from neural data. Phys Rev E 66:051903. 10.1103/PhysRevE.66.051903 12513519

[B100] Victor JD (2006) Approaches to information-theoretic analysis of neural activity. Biol Theory 1:302–316. 10.1162/biot.2006.1.3.302 19606267 PMC2709861

[B101] Victor JD, Purpura KP (1996) Nature and precision of temporal coding in visual cortex: a metric-space analysis. J Neurophysiol 76:1310–1326. 10.1152/jn.1996.76.2.1310 8871238

[B102] Vinga S (2013) Information theory applications for biological sequence analysis. Brief Bioinfo 15:376–389. 10.1093/bib/bbt068 24058049 PMC7109941

[B103] Wibral M, Lizier JT, Priesemann V (2014a) Bits from brains for biologically inspired computing. Front Robotics AI 2:1–25. 10.3389/frobt.2015.00005

[B104] Wibral M, R Vicente, Lizier JT, eds (2014b) Directed information measures in neuroscience. Berlin: Springer.

[B105] Wibral M, Pampu N, Priesemann V, Siebenhühner F, Seiwert H, Lindner M, Lizier JT, Vicente R (2013) Measuring information-transfer delays. PloS One 8:10.1371/journal.pone.0055809 PMC3585400 23468850

[B106] Williams PL, Beer RD (2010) Nonnegative decomposition of multivariate information. arXiv 1004.2515.

[B107] Williams PL, Beer RD (2011) Generalized measures of information transfer. arXiv 1102.1507.

[B108] Wollstadt P, Martinez-Zarzuela M, Vicente R, Diaz-Pernas FJ, Wibral M (2014) Efficient transfer entropy analysis of non-stationary neural time series. arXiv 1401.4068. 10.1371/journal.pone.0102833 PMC4113280 25068489

[B109] Wollstadt P, Sellers KK, Rudelt L, Priesemann V, Hutt A, Fröhlich F, Wibral M (2017) Breakdown of local information processing may underlie isoflurane anesthesia effects. PLoS Comput Biol 13:e1005511. 10.1371/journal.pcbi.1005511 28570661 PMC5453425

[B110] Wolpert DH, Wolf DR (1995) Estimating functions of probability distributions from a finite set of samples. Phys Rev E 52:6841. 10.1103/PhysRevE.52.6841 9964199

[B111] Zar JH (2010) Biostatistical Analysis, 5th Edition Upper Saddle River, NJ: Prentice Hall, Inc.

