# Supplementary material for: A Tutorial for Information Theory in Neuroscience
Source: eNeuro. 2018 Sep 11;5(3):ENEURO.0052-18.2018. doi: 10.1523/ENEURO.0052-18.2018 (PMC6131830; doi:10.1523/ENEURO.0052-18.2018)
Supplement: Extended Data — Download Extended Data, ZIP file [file sup_enu-eN-REV-0052-18-s02.zip › Neuroscience Information Theory/ToolboxReadMe.pdf]

%%
%%%%%%%%%%%%%%%%%%   Neuroscience Information Theory Matlab Toolbox   %%%%%%%%%%%%%%%%%
%%

This toolbox contains software to perform numerous information theory analyses. The software is designed primarily with neuroscience applications in mind, but it could easily be applied to data from other research areas. Questions, comments, suggestions, or bug reports should be directed to REDACTED.

We divide this README file into several important sections:

- Getting Started
- Quick Start/Use Guide
- Fully Customizable Analysis Guide
- Demos
- Detailed Function Descriptions
- Simulations

%%
%%% Getting Started %%%

If you are running this code by installing the Matlab toolbox, then you should simply install the toolbox (Neuroscience Information Theory.mlbtx) by right clicking on it and clicking install, if you haven't installed it already.

If you simply downloaded all the code, you should add the main directory (Neuroscience Information Theory) to the Matlab search path. This will ensure that all the demos and simulations will run correctly.

%%
%%% Quick Analysis Guide %%

If you're anything like me, you probably want to jump right in to the analysis. Though the software is highly customizable (see below for details), we have produced several macros to allow the user to quickly and easily analyze their data. The format and type requirements for these functions are very minimal. Any type of data, including continuous or discrete and trial based or single trial data can be analyzed easily. These functions contain customization features that we anticipate will be commonly used (e.g. variable number of discretization states, variable delays, variable significance testing), but they do not possess the full range of customization that is available via the whole software suite. The quick functions included in the software package along with their functionality are listed below (type help FunctionName to see details about the use of each function):

quickent – Calculates the entropy of a variable

quickMI – Calculates the mutual information between two variables

quickTE – Calculates the transfer entropy between two variables

quickPID – Calculates the partial information decomposition for three variables

In addition to the demos described below, these functions serve as a good starting point to understanding the whole analysis.

%%
%%% Fully Customizable Analysis Guide %%%

The full analysis suite contains several versatile functions that can be used individually or combined by the user into a custom function to suit his or her analysis needs. In general, utilizing the software proceeds by the following steps (see demo scripts below for demonstrations of this process):

1. Get raw data into Matlab.
2. Reformat the data for processing.
3. Convert the data to discrete states (we will refer to this as “stating” the data, similar to “binning”).
4. Calculate the information quantity of interest.

%%%
Step 1: Get raw data into Matlab.

To a large extent, this step is up to the user to negotiate. Given the wide range of data gathering systems and data formats, it is not practical for us to accommodate all possible variations. Typically, though, this process is not difficult. All that is necessary is to obtain a Matlab vector or array that contains the data as double real numbers (e.g. voltage, position, BOLD intensity, etc.) or as integers (e.g. stimulus type, spike times, etc.).

%%%
Step 2: Reformat the data for processing.

Note, the function formattool is especially helpful in performing the reformatting discussed below. Type ‘help formattool’ for assistance.

An important concern in this analysis the role of time and the use of time bins. Below we will discuss software tools that can be used to easily bin or rebin the data, but it requires comment first that the data must be binned in time. Since experimental data is usually binned at the sampling frequency for the recording system, the binned character of the data is assumed.

The analysis software assumes a specific format which we will now describe. All data to be processed should be put in a Matlab array or cell array called DataRaster. The DataRaster format accommodates a wide range of data organization needs. First, if different types of data are available, the user can decide to use a cell array for DataRaster to store different types of data (referred to as “data categories”). In this case, DataRaster should be a number of data categories by 1 cell array. This can be especially useful for storing data with different numbers of variables, data with different numbers of time bins, or simply to help the user keep different types of data separate.

Data from each data category should be organized as a double array. This array should have dimension number of variables by number of time bins by number of trials. If only one data category is being utilized, DataRaster can be a double array with this format. Alternatively, data for each data category should be stored as this double array in the corresponding element of the DataRaster as a cell array.

For example, if the 3rd variable in data category 2 produced a value of 5.43 on the 8th trial in the 12th time bin, then DataRaster{2}(3,12,8) = 5.43. If there were only one data category in DataRaster and the 2nd variable produced a value of 3.29 on the 5th trial in the 10th time bin, then DataRaster(2,10,5) = 3.29.

Note that the different data categories can have different numbers of variables. They can also have different numbers of time bins (except in single trial analyses). However, for any information theory analysis to occur involving variables between categories, those categories must have matching numbers of trials or, if only single trial data is used, matching numbers of time bins.

%%%
Step 3: Convert the data to discrete states (we will refer to this as “stating” the data, similar to “binning”).

Once the data are formatted in the previous step into DataRaster, the data are converted into discrete states using the function data2states. This function can utilize several different methods for discretization and produces as an output a StatesRaster structure. The StatesRaster has the same type and dimensions as the DataRaster, but the data will be converted to integer states. Type 'help data2states' for a complete description of the discretization methods. Also, symbolic stating methods (states based on data value ranking order) can be implemented using the function symbolicdata2states. Type 'help symbolicdata2states' for a complete description of this process.

Following stating the data, additional restating can be conducted to combine adjacent time bins using word states via the function wordstates. Word states represent specific ordered combinations of states. For instance, the appearance of state 1 at time t and state 2 at time t+1 may be relevant to the system beyond simply the appearance of state 1 at time t alone and state 2 at time t+1 alone. Note that this restating will reduce the number of time bins. Type 'help wordstates' for a complete description of this function.

%%%
Step 4: Calculate the information quantity of interest.

The information theory analysis is carried out by the function instinfo. It takes as its input the StatesRaster and information about the type of information theory analysis to perform. Note that in the case of trial based data, the information analysis is performed across trials at a given time (i.e. time locked points across trials are used as the observations in the joint probability distribution estimation). This will produce information results as a function of time throughout the trial. In the case of single trial data, the information analysis is performed across time bins (i.e. points through time are used as the observations in the joint probability distribution estimation). This will produce a single information result for the whole recording. Type 'help instinfo' for a complete description of the analysis methods available and how to utilize the function.

%%
%%% Demos %%

Included in the software package are several demo scripts which contain numerous short demonstrations of various features of the software. In the table below, we list the most important features of each demo to aid the user to finding the most helpful section of the code. Note, AllDemos can be called in its entirety to run all of the demos, but it is often more helpful to run individual demos. Suggestions for additional demos are encouraged (REDACTED).

Demo	Information Theory Measure	Discrete or Continuous?	Single Trial or Trial Based?	Number of Variables	Significance Testing	Discretization Method	Notes
Ent1	Entropy	Continuous	Trial Based	1	N/A	Uniform Width Bins	
Ent2	Entropy	Discrete	Single Trial	1	N/A	Uniform Count Bins	
Ent3	Entropy	Continuous	Trial Based	6	N/A	Uniform Width Bins	Demonstrates Data Category Formatting
JEnt1	Joint Entropy	Discrete	Single Trial	3	N/A	Uniform Count Bins	
CEnt1	Conditional Entropy	Continuous	Trial Based	2	N/A	Uniform Width Bins	
MI1	Mutual Information	Discrete	Trial Based	2	Off	N/A	

MI2	Mutual Information	Continuous	Single Trial	3	On	Uniform Count Bins	Demonstrates Interaction On/Off with p-value
JMI1	Joint Mutual Information	Discrete	Single Trial	4	On	Uniform Width Bins	
TEnt1	Transfer Entropy	Continuous	Trial Based	2	On	Uniform Count Bins	
TEnt2	Transfer Entropy	Discrete	Single Trial	2	On	Uniform Count Bins	Demonstrates TE Calculation for Neuron Action Potentials
PID1	Partial Information Decomposition	Discrete	Trial Based	3	On	N/A	
MI3	Mutual Information	Continuous	Single Trial	3	On (w/ Null Model)	Uniform Count Bins	Demonstrates the Use of a Predefined Null Model

%%
%%% Detailed Function Descriptions %%%

All functions are well documented. For assistance with any function, type 'help FunctionName' for a complete description of the function and how to use it.

%%
%%% Simulations %%

In addition to the simple general demos that are described above, we also include numerous neuroscience oriented simulations stored in a subdirectory to the toolbox called "simulations." The simulations are run using the Matlab scripts neuroDemoX, where X runs from 1 to 5. The other functions in the simulations subdirectory are various models and other support programs.

The simulations produce pdf figure panels that are identical to figures published with the toolbox. Note that small changes in results may be observed due to stochastic fluctuations.
