# Supplementary material for: A Tutorial for Information Theory in Neuroscience
Source: eNeuro. 2018 Sep 11;5(3):ENEURO.0052-18.2018. doi: 10.1523/ENEURO.0052-18.2018 (PMC6131830; doi:10.1523/ENEURO.0052-18.2018)
Supplement: Extended Data Figure 7-1 — Download Figure 7-1, DOCX file [file sup_enu-eN-REV-0052-18-s01.docx]

| $Y$ | $X_{1}$ | $X_{2}$ | Converging Redundant | Diverging Redundant | Converging Synergistic | Diverging Synergistic | Converging Unique | Diverging Unique |
| --- | --- | --- | --- | --- | --- | --- | --- | --- |
| 0 | 0 | 0 | 0.5 | 0.5 | 0.25 | 0.125 | 0.25 | 0.125 |
| 0 | 0 | 1 | 0 | 0 | 0 | 0 | 0.25 | 0.125 |
| 0 | 1 | 0 | 0 | 0 | 0 | 0 | 0 | 0 |
| 0 | 1 | 1 | 0 | 0 | 0.25 | 0.125 | 0 | 0 |
| 1 | 0 | 0 | 0 | 0.25 | 0 | 0.125 | 0 | 0 |
| 1 | 0 | 1 | 0 | 0 | 0.25 | 0 | 0 | 0 |
| 1 | 1 | 0 | 0 | 0 | 0.25 | 0 | 0.25 | 0.125 |
| 1 | 1 | 1 | 0.5 | 0.25 | 0 | 0.125 | 0.25 | 0.125 |
| 2 | 0 | 0 | 0 | 0 | 0 | 0 | 0 | 0.125 |
| 2 | 0 | 1 | 0 | 0 | 0 | 0.125 | 0 | 0 |
| 2 | 1 | 0 | 0 | 0 | 0 | 0.125 | 0 | 0.125 |
| 2 | 1 | 1 | 0 | 0 | 0 | 0 | 0 | 0 |
| 3 | 0 | 0 | 0 | 0 | 0 | 0 | 0 | 0 |
| 3 | 0 | 1 | 0 | 0 | 0 | 0.125 | 0 | 0.125 |
| 3 | 1 | 0 | 0 | 0 | 0 | 0.125 | 0 | 0 |
| 3 | 1 | 1 | 0 | 0 | 0 | 0 | 0 | 0.125 |

**Figure 7-1: Joint probability distributions for the examples shown in Figure 7.**
